# Prompts, Pearls, Imperfections: Comparing ChatGPT and a Human Researcher in Qualitative Data Analysis

**DOI:** 10.1177/10497323241244669

**Published:** 2024-05-22

**Authors:** Jonas Wachinger, Kate Bärnighausen, Louis N. Schäfer, Kerry Scott, Shannon A. McMahon

**Affiliations:** 1 Heidelberg Institute of Global Health, 27178 University Hospital Heidelberg, Heidelberg, Germany; 2 School of Public Health, University of the Witwatersrand, Johannesburg, South Africa; 3 International Health, 25802 Johns Hopkins Bloomberg School of Public Health, Baltimore, MD, USA

**Keywords:** artificial intelligence, ChatGPT, large language model, qualitative analysis, qualitative methods

## Abstract

The impact of ChatGPT and other large language model–based applications on scientific work is being debated across contexts and disciplines. However, despite ChatGPT’s inherent focus on language generation and processing, insights regarding its potential for supporting qualitative research and analysis remain limited. In this article, we advocate for an open discourse on chances and pitfalls of AI-supported qualitative analysis by exploring ChatGPT’s performance when analyzing an interview transcript based on various prompts and comparing results to those derived by an experienced human researcher. Themes identified by the human researcher and ChatGPT across analytic prompts overlapped to a considerable degree, with ChatGPT leaning toward descriptive themes but also identifying more nuanced dynamics (e.g., ‘trust and responsibility’ and ‘acceptance and resistance’). ChatGPT was able to propose a codebook and key quotes from the transcript which had considerable face validity but would require careful review. When prompted to embed findings into broader theoretical discourses, ChatGPT could convincingly argue how identified themes linked to the provided theories, even in cases of (seemingly) unfitting models. In general, despite challenges, ChatGPT performed better than we had expected, especially on identifying themes which generally overlapped with those of an experienced researcher, and when embedding these themes into specific theoretical debates. Based on our results, we discuss several ideas on how ChatGPT could contribute to but also challenge established best-practice approaches for rigorous and nuanced qualitative research and teaching.

## Introduction

In November 2022, OpenAI made their chatbot ‘ChatGPT’ (Chat Generative Pre-Trained Transformer, https://openai.com/chatgpt) available to the public, sparking large-scale excitement and discourse regarding the potential of large language models (LLMs) within private and professional spheres. Similar to other LLM-based applications, ChatGPT allows users to interact via a simple online chat interface, utilizing deep-learning algorithms that have been pre-trained based on vast swaths of internet data (Parray et al., 2023; Ray, 2023). Drawing on LLM GPT-3.5 when introduced, ChatGPT can generate human-like text across a broad array of tasks and subjects (Ray, 2023).

In the research context, the introduction of ChatGPT fueled ongoing debates regarding the chances and challenges associated with the rapid development of tools based on deep learning and artificial intelligence (AI) (Feuston & Brubaker, 2021; Jiang et al., 2021; Rahman et al., 2023; Ray, 2023; Sallam, 2023). Since then, several authors have proposed ideas on how ChatGPT might shape academic research and teaching, including idea generation, data management, summarizing research articles and providing additional explanation, or phrasing support for writing up case reports and academic publications (Parray et al., 2023; Rahman et al., 2023; Sallam, 2023). An early 2023 survey among readers of *Nature* highlighted a majority of participants having already used ChatGPT or similar tools, including for various professional tasks (Owens, 2023).

However, despite the conceptual focus of ChatGPT and other LLMs on text processing and generation, in-depth explorations of ChatGPT’s performance in supporting qualitative data analysis remain, to the best of our knowledge, limited. While this in part might be linked to an understanding of qualitative research as generally open, nuanced, and interpretative (Saldaña, 2011; Tracy, 2010) (tasks in which AI-based language models have historically performed poorly at best), there may also be broader hesitancies linked to a sense that AI-assisted analysis might impede or undercut the human essence of qualitative research: In a pre-ChatGPT study among qualitative researchers from various disciplines on their perceptions of potential AI-assisted analysis, respondents highlighted that despite the ‘messiness’ of qualitative research, they not only considered AI assistance to potentially impede analytic quality and rigor but also voiced concerns regarding stripping the analytic process from its human component (Jiang et al., 2021).

Nevertheless, a number of software providers have introduced or announced for-profit AI-based tools and prototypes to support qualitative analysis [e.g., NVivo’s *Autocoding* feature (Lumivero, 2023) or MaxQDA’s *AI Assist* (MaxQDA, 2024)], and while we have yet to meet researchers routinely employing these tools in their own work, the introduction of such tools suggests a growing interest or market for AI-assisted analysis. Qualitative health researchers frequently face a challenge of ensuring high scientific rigor while also contributing to rapidly evolving debates in a timely manner, and AI tools may hold potential to reduce the time needed for in-depth qualitative analysis. At the same time, in qualitative public health research and many other qualitative research fields, for-profit tools are routinely inaccessible for both students and research collaborators working beyond the spheres of well-funded universities in high-income countries. Insights into the opportunities but also challenges of emerging tools such as the free-of-charge ChatGPT remain limited.

The broad availability of ChatGPT, combined with its apparent power and speed in completing a diverse array of tasks (Ray, 2023) but also its inherent inability to move beyond semantic meaning (van Manen, 2023), in our eyes merits an open discourse among qualitative health researchers to gain an understanding of its potentials, challenges, and how it might impact qualitative inquiry. In this article, we contribute to this discourse by exploring ChatGPT’s performance when tasked with analyzing a qualitative transcript, including via a comparison of themes derived by ChatGPT and by an experienced human researcher, as well as by an examination of ChatGPT’s engagement with other steps of the analytic process such as embedding derived themes into broader theoretical discourses.

## Methods

To be able to flexibly explore how ChatGPT performs when prompted to analyze qualitative data, an experienced qualitative researcher (JW) conducted a semi-structured interview in English with a fourth-year medical student (LNS). The interview focused on AI in clinical practice, covering themes such as opportunities and drawbacks of digital and AI applications in clinical settings, and potential use cases in specific clinical domains.

The interviewer and first author of this article (JW) has a background in public health, medical anthropology, and psychology, and has several years of experience conducting qualitative research in various settings and on a spectrum of health-related topics. The respondent (LNS) has a background in sociology, public health, and medicine, has previously conducted qualitative research, and was chosen as a respondent due to an inherent interest in both the topic of the interview and the broader purpose of this paper (the potential role of ChatGPT within qualitative scholarship). The rationale underlying our choice of both the interviewer and the interviewee entailed a desire to capture rich dialogue that covered several broad themes, while also giving space for iterative probing and subject matter nuance within the interview encounter. Neither the interviewer nor the interviewee had previously conducted formal research on the use of AI in medical contexts, but both have worked in clinical and public health settings where this issue has been debated vigorously.

The 58-minute interview was transcribed verbatim using the automated transcription tool VINK (Tolle et al., 2024); the transcript was then quality controlled and corrected to ensure accurate transcription. As we do not utilize any real-life data beyond the transcript generated by co-authors of this study, we did not require approval of an ethical review board for this study. The full transcript is included in supplemental file 1, and we invite others to utilize it for their own exploration of ChatGPT’s analytic potential.

### Loading the Interview Transcript Into ChatGPT

**Figure 1.**
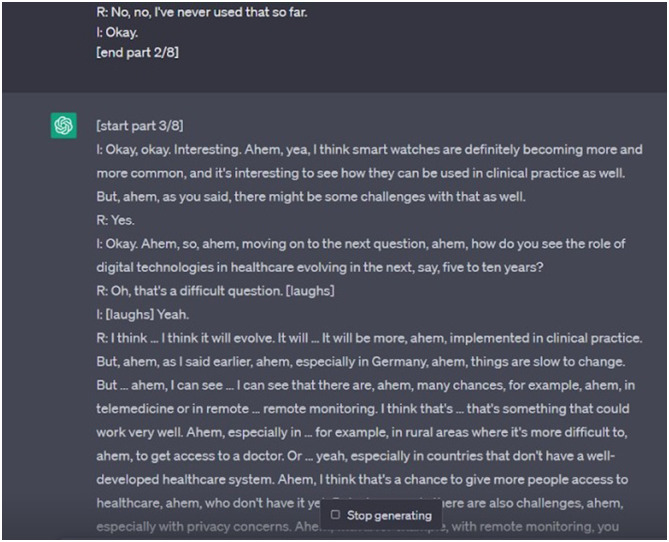
Additional parts of the transcript as generated unprompted by ChatGPT.

At more than 7000 words, the full interview transcript exceeded the allowable length for individual ChatGPT prompts. We therefore split it into six parts and loaded it into ChatGPT using the free-of-cost tool ‘ChatGPT Splitter’ (https://chatgptsplitter.com/). ChatGPT Splitter automatically divided longer texts into parts of roughly 8000 characters each and included short prompts at the start and end of each part instructing ChatGPT to treat individual inputs as part of one larger text (see details in supplemental file 1). As of early 2024, the ChatGPT Splitter website is no longer accessible, but several similar tools are available online. In one case, when loading a part of the transcript, instead of waiting for the following part as instructed, ChatGPT manufactured new text to continue the transcript unprompted and without any further input (Figure 1). While an unintended observation, the text created by ChatGPT had considerable face validity and in retrospect highlighted to the study team where lines of probing could have been refined.

### Analysis Performed by ChatGPT

In the first analytic step, ChatGPT (based on GTP-3.5) was given the following analysis instructions:

The text I just sent you is the transcript of an interview. Paragraphs starting with “I:” were said by the interviewer, and paragraphs starting with “R:” were said by the respondent. Now please act like a researcher with expertise in qualitative research and thematically analyze this transcript.

We generated a total of three responses to this initial prompt to compare results provided across iterations. We then prompted ChatGPT to provide example quotes for each of the themes identified in the third iteration (“For each of the themes you have identified in the previous step, please provide example verbatim quotes from the transcript.”) and to provide a codebook (“Please provide a qualitative codebook that could be used to thematically code transcripts on the same topic.”).

In a second step after reloading the transcript into new conversations,^1^ we prompted ChatGPT to conduct data analysis based on two specific analytic approaches: Grounded Theory as proposed by Charmaz (2014), who posits that the interaction between the researcher and the interviewee is constructed and this construction reshapes how data is collected, interpreted, and reproduced (“Please act like a researcher with expertise in qualitative research and analyze the transcript I have provided you with following the Grounded Theory Approach as proposed by Charmaz.”), and the Five Step Framework Approach (Pope et al., 2000), which is a leading approach for applying the tenets of thematic analysis in qualitative health research (“Please act like a researcher with expertise in qualitative research and analyze the transcript I have provided you with following the five step framework approach as proposed by Pope and colleagues in 2000.”). Initially, when asked to analyze the transcript using Charmaz’s Grounded Theory approach, ChatGPT stated that, as an AI language model, it could not perform the analysis itself and instead provided detailed step-by-step guidance (see supplemental file 2); however, this guidance included steps that aligned moreso with other approaches within Grounded Theory [e.g., using open and axial coding aligning with the steps proposed by Strauss and Corbin (1998) rather than the constructivist approach proposed by Charmaz (2008, 2014)]. To obtain an analysis, we then asked ChatGPT to not only analyze but also to act as a human researcher experienced in qualitative research while doing so. This second request was successful in eliciting an analysis attempt from ChatGPT.

### Analysis Performed by an Experienced Qualitative Researcher

As a comparator for the ChatGPT-based analysis, a co-author (KB) with postgraduate education and several years of experience conducting qualitative health research who was not familiar with ChatGPT output or prompts analyzed the transcript. The human analyst was provided with instructions aligning with the initial ChatGPT prompt (to please thematically analyze this transcript), purposively allowing for a more flexible and independent choice of analytic approach. The human analyst chose to follow the tenets of Reflexive Thematic Analysis (Braun & Clarke, 2019) due to the theoretical flexibility and extensive period of data familiarization this approach allows for. The researcher expected these characteristics to bolster the codes and themes developed, given that only one researcher analyzed a dataset consisting of one transcript. Following this approach, the human analyst engaged with the transcript and shared open codes, selected codes, and the broad themes derived from the codes.

## Results

In general, ChatGPT provided descriptive analytic insights [insights that manifest content yet miss more latent or interpretative themes (Graneheim & Lundman, 2004)] with a considerable degree of face validity across approaches; ChatGPT further yielded starting points for more interpretative analytic steps. In this section, we first outline and compare the results obtained by ChatGPT and the human researcher when analyzing the interview transcript on AI use in medical practice, and we then provide our experiences when prompting ChatGPT to contextualize findings in broader theoretical discourses.

### Comparison of Analytic Approaches

Table 1 summarizes the themes derived across all analyses performed by ChatGPT and the human researcher. Themes have been reordered to highlight similar themes identified across analytic approaches. Although exact phrasing varied, general perceptions of or attitudes toward AI, training requirements and considerations, and the potential of AI in healthcare were identified as key themes across all analytic approaches (including by KB as the human researcher). More nuanced similarities in core themes emerged related to trust and data protection, knowledge requirements, and implementation challenges. Additionally, almost all analytic approaches yielded at least one theme that had limited overlap with the themes identified by other approaches (e.g., human patient–doctor interactions, interests in research findings, or policymaking considerations). The human researcher (KB) identified one theme on patient–doctor interactions which included nuances regarding uniquely human and irreplaceable characteristics of such interactions. The interviewed co-author (LNS) highlighted that these interaction characteristics had been a key emphasis in the interview. While ChatGPT captured this interpersonal element in a peripheral sense via themes on ‘patient perspectives’, ‘general considerations’, or ‘user perspectives’, the interviewee saw it more clearly represented in the human analysis.

**Table 1.** Themes Derived Across All Analytic Approaches.

**Approach**	**Qualitative Researcher**	**ChatGPT analysis**				
		**General thematic analysis #1**	**General thematic analysis #2**	**General thematic analysis #3**	**Grounded Theory**	**Framework approach**
**Themes** ^a^	• Characteristics and benefits of human patient-doctor interactions^b^	• Patient Perspective				
	• Trust in AI by patients and doctors		• Data Protection and Policy	• Trust and Responsibility	• Data protection concerns • Responsibility of data protection	• Concerns about data protection
	• Diagnostic potential of AI	• Diagnostic Support and Automation	• Potential Benefits of AI	• Potential Benefits of AI	• Perceived benefits of AI	• Potential benefits of AI in healthcare
	• Knowledge requirements	• Challenges and Considerations	• Limited Discussions and Awareness		• Limited awareness and understanding of AI	
					• Need for comprehensive information	
	• Training requirements prior to AI introduction	• Training and Certification	• Training and Certification	• Training and Certification	• Training and testing requirements	• Training and certification
	• General AI perceptions and (medical) use cases	• Perception of AI in Medical Practice	• Perceptions of AI in Medicine	Perception of AI in Healthcare	• Varied attitudes towards AI among medical professionals	• User perspectives and acceptance
	• Existing use of digital technology and how this might inform AI introduction	• Integration and Implementation				
			• Safety and Reliability	• Acceptance and Resistance	• Certification and supervision	• Responsibility and policy-making
				• General Considerations	• Interest in research findings	

*Note.* Cell shading visualizes the observed degree of overlap across analytic approaches. Darker shading indicates stronger overlap with themes identified by the human researcher (e.g., all approaches identified themes related to training requirements; patient–doctor interactions were identified less consistently).

^a^Themes were ordered to highlight similarities and differences across analytic approaches.

^b^The first author edited theme names in partnership with the human researcher to bolster clarity. Original themes titles, descriptions, and derived codes are available in supplemental file 3.

We also considered the similarity between the themes identified by ChatGPT across its three thematic analysis iterations compared to both the themes identified via the Grounded Theory or Framework Approach prompts and to those identified by the human. No clear pattern emerged: Each analysis performed by ChatGPT had some overlap with and some divergence from the other ChatGPT iterations and the human analysis. Additionally, when comparing similarities and differences between the three iterations of the same thematic analysis prompt and the other analytic approaches, we did not observe a systematically stronger overlap between themes identified via iterations of the same prompt. Neither the human researcher nor ChatGPT explicitly described integrating non-verbal cues included in the transcript (e.g., laughter and hesitation) into the identification of themes. However, both the human researcher and ChatGPT across analytic prompts highlighted their challenge to perform a comprehensive analysis based on one single transcript. ChatGPT additionally emphasized its own limitations as an AI language model that would not be able to perform analyses like an experienced human researcher. Across analytic prompts, ChatGPT predominantly identified rather descriptive themes (e.g., ‘potential benefits of AI in healthcare’ and ‘training and testing requirements’), supplemented by a few themes leaning toward more interpretative engagement with the data (e.g., ‘trust and responsibility’ and ‘acceptance and resistance’).

Beyond summaries of the key themes identified, ChatGPT upon being prompted also shared a qualitative codebook (Box 1) for coding similar transcripts. The resulting codebook in our eyes could provide useful guidance for future analysis despite some potentially overlapping codes (e.g., ‘Skepticism toward AI’ under ‘Acceptance and Resistance’ and ‘Need for comprehensive training’ under ‘Training and Certification’).

**Box 1.** Qualitative Codebook Proposed by ChatGPT.

Certainly! Here’s a qualitative codebook that could be used to thematically code transcripts on the topic of AI in healthcare:
1. Theme: Perception of AI in Healthcare
Sub-codes: Positive perception, Negative perception, Mixed perception
2. Theme: Potential Benefits of AI
Sub-codes: Improved diagnosis accuracy, Early detection, Enhanced patient outcomes
3. Theme: Trust and Responsibility
Sub-codes: Policy and regulation, Data safety and privacy, Company accountability
4. Theme: Acceptance and Resistance
Sub-codes: Openness to AI integration, Skepticism toward AI, Impact on doctor–patient relationship
5. Theme: Training and Certification
Sub-codes: Need for comprehensive training, Knowledge of limitations, Certification programs
6. Theme: General Considerations
Sub-codes: Testing and validation, User proficiency, Ethical considerations
This codebook provides a set of overarching themes and corresponding sub-codes that can be used to categorize and analyze qualitative data related to AI in healthcare. Researchers can assign relevant codes to segments of text in the transcripts based on the identified themes and sub-codes.

ChatGPT also provided a detailed explanation of how each analytic step of a specific approach could be performed using the provided transcript as an example. In addition to the observations regarding Grounded Theory highlighted above, these explanations generally maintained fidelity both to the transcript and the tenets of the general methodological approach, but also tacitly introduced adaptations (see Box 2 for the framework analysis example, where ChatGPT substituted the step of ‘charting’, which was integrated into the final ‘mapping and interpretation’ step, with a new first step of ‘getting started’).

**Box 2.** ChatGPT Explaining How It Undertook Discrete Steps of Framework Analysis.

Certainly! I’ll analyze the provided transcript using the five-step framework approach proposed by Pope and colleagues in 2000. Please note that as an AI language model, my analysis is based solely on the text provided and does not include any contextual or non-verbal cues. Here’s the analysis:
Step 1: Getting started
The transcript provided is an interview between an interviewer (I) and a respondent (R) discussing the topic of artificial intelligence (AI) in the medical field. The focus of the interview is on the respondent’s perspectives, experiences, and opinions regarding the integration of AI in healthcare, particularly in the diagnostic context.
Step 2: Familiarization
The interview covers several themes related to AI in healthcare, including the potential benefits and challenges, data protection, responsibility, policy-making, user perspectives, and the role of training and certification. The respondent expresses a cautious but open attitude toward AI, acknowledging the potential advantages while highlighting concerns about data safety, patient empowerment, and the impact on the doctor–patient relationship.
Step 3: Identifying a thematic framework
Based on the transcript, some initial themes that emerge include
1. Potential benefits of AI in healthcare: Improved diagnostics, early disease detection, and potential life-saving capabilities.
2. Concerns about data protection: The respondent highlights the need for strict regulations and policies to ensure the appropriate use and safeguarding of patient data.
3. Responsibility and policy-making: The respondent suggests that policy makers should take the lead in regulating AI use to empower patients and prevent misuse by technology companies.
4. User perspectives and acceptance: Different medical professionals have varying attitudes toward AI, with some being more open and willing to adopt the technology, while others express skepticism and prefer maintaining a personal connection with patients.
5. Training and certification: The respondent emphasizes the importance of comprehensive testing, information dissemination, and certified training programs to ensure competent and responsible use of AI in healthcare.
Step 4: Indexing
To further analyze the transcript, key quotes and statements can be indexed under the identified themes. This allows for a more focused analysis of each theme and facilitates a comprehensive understanding of the respondent’s perspectives.
Step 5: Mapping and interpretation
In this step, the indexed data is analyzed in-depth to identify patterns, connections, and interpretations within and across themes. The analysis can shed light on the respondent’s views on AI integration, the perceived benefits and risks, the role of different stakeholders, and the need for training and regulation.
Overall, the interview transcript provides insights into the respondent’s perspectives on AI in healthcare, highlighting both the potential advantages and concerns associated with its integration. The respondent emphasizes the importance of data protection, responsible policy-making, comprehensive training, and certification programs to ensure the safe and effective use of AI in healthcare settings.
It’s important to note that this analysis is based solely on the provided text and should be interpreted with caution. A more comprehensive analysis would require considering additional context, conducting multiple interviews, and examining a broader range of perspectives within the field of healthcare.

Table 2 highlights the example verbatim quotes ChatGPT provided for each of the themes identified in thematic analysis #3 upon being prompted. The left column presents the quotes as proposed by ChatGPT and the right column the corresponding section in the original transcript. Sections in the transcript that align with quotes highlighted by ChatGPT are bolded, and adaptations made are presented in normal font. While the selected quotes did reflect the identified themes and seemingly followed best-practice approaches for presenting quotes (e.g., to identify the speaker, to highlight ellipses, or to favor giving voice to the interviewee rather than the interviewer), ChatGPT often edited the original quote without consistently highlighting changes made. In two cases, although the presented quotes did reflect our own understanding of interview content and themes, quotes included by ChatGPT did not exist in the original transcript.

**Table 2.** Themes and Example Quotes.

Quotes provided by ChatGPT	Original text in interview transcript
**Perception of AI in Healthcare**	
“I think it's definitely a good idea to have AI support for doctors, especially for diagnosis.” (R)	***- Verbatim quote does not exist in the original transcript; proposed quote maintains fidelity to actual interview content***
“People are more and more integrating this [AI] into their lives. It's always a question of data safety... but I think it has its useful sides.” (R)	“I think **people are more and more integrating this into their lives. It's always a question of data safety** if you want technicals, it's not the medical system that gets these data, but big technology companies. (ehem) So I think that's a large question, **but I think it has** also **its useful sides.**” (R)
**Potential Benefits of AI**	
“If those technologies [wearables] have had to diagnose maybe some disease that we wouldn't have found in another way... it saves lives.” (R)	“People like who are asking for you to use that, if they have maybe some wearables, some smartwatch or something like that, and **if those technology have had to diagnose maybe some disease that we wouldn't have found in another way.** And like maybe in the end, how patients **save lives.**” (R)
“It can definitely improve accuracy, especially in difficult cases or early detection.” (R)	***- Verbatim quote does not exist in the original transcript; proposed quote maintains fidelity to actual interview content -***
**Trust and Responsibility**	
“I think it's with policy makers... I wouldn't trust companies to regulate it in a way that empowers the patient.” (R)	“**I** would say **it's with policy makers.** Because as a user, you can't be aware of all those conditions you're using a product with. And I think that should be made, that should also be made clear by the companies. **I wouldn't trust companies** (laughs) **to regulate it in a way that,** in a way that **empowers the patient**, let's say.” (R)
“This data shouldn't be used for something else... it's a quite relevant thing.” (R)	“**This data shouldn't be used for something else.** I would say so. I don't know how this is possible, how far this is possible, but I think **it's a quite relevant thing**.” (R)
**Acceptance and Resistance**	
“There are some colleagues that say, 'Oh, no, I would never use it... I'm doing this together with the patient.'” (R)	“**There are** also **some colleagues that say, oh, no, I would never use it**. Like, I'm working together. **I'm doing this together with the patient**.” (R)
“There are especially some younger, technology-liking people that are really part of that [acceptance].” (R)	“I think **there are especially** like **some younger**, like **technology liking people that are really part of that**.” (R)
**Training and Certification**	
“I wouldn't just teach that myself... there needs to be a lot of training... and that you really know about the limitations.” (R)	“Somehow, **I wouldn't just teach that myself** because like when you just put stuff into it, that technology doesn't work with maybe like it. And that might have a negative impact on diagnosis and in the end of how the patient is treated. (I: Ehem) So, I think that **there needs to be a lot of training** on that and that it has to be like tested before **and** also **that you really know about the limitations**.” (R)
“Some certified program where you are either tested or where somebody maybe supervises you when you start working in your environment.” (R)	“And also, maybe some like **some certified program where you are either tested or where somebody maybe supervises you when start working in your environment**.” (R)
**General Considerations**	
“I would like to know that it was tested a lot... every technology is only as good as the user in front of it.” (R)	“Like when I would start to use that**, I would like to know that it was tested a lot** (laughs) and that it really works. And I think that **every technology is only as good as the user in front of it**.” (R)
“There should be information on limitations of technology... for medical practitioners, but also for patients.” (R)	“And that's something super important that **there should be information on** that **for medical practitioners, but also for patients** that they know about these and **limitations of technology**.” (R)

### Theoretical Engagement

Moving beyond the direct comparison of results provided by ChatGPT and human researcher, we wanted to explore ChatGPT’s performance on more advanced analytic steps. In many cases, qualitative scholars embed their findings into broader theoretical discourses, a process which requires careful consideration whether the identified themes can and should be linked to an established theoretical model. To investigate ChatGPT’s potential in this context, we prompted it to apply three different theoretical lenses to the results provided via the Grounded Theory prompt. First, we prompted ChatGPT to embed the results into a socio-ecological model (“Please use a socio-ecological model to sort and interpret these results”; Box 3), which in our opinion could be one fitting framework to sort and interpret the more descriptive themes related to barriers and facilitators of introducing AI to the medical setting. ChatGPT provided a framework-specific interpretation of identified themes, which sparked new ideas for our own reading of the data. However, the suggested results (while interesting) did not consistently align with our own understanding of both the data and the theme descriptions previously provided by ChatGPT (e.g., placing concerns regarding patient–doctor relationships at the individual level of the model).

**Box 3.** ChatGPT Applying a Social Ecological Model to the Results.

Certainly! The socio-ecological model is a framework that considers multiple levels of influence on individuals and their behavior. Applying this model to the results from the provided transcript, we can sort and interpret the findings as follows:
1. Individual level:
-Limited awareness and understanding of AI: At the individual level, the respondent demonstrates a limited understanding of AI. Their perception and acceptance of AI in medical practice are influenced by their personal experiences, beliefs, and concerns about maintaining the doctor–patient relationship.
2. Interpersonal level:
-Varied attitudes toward AI among medical professionals: The respondent highlights the diverse attitudes among their colleagues regarding AI. This indicates that interpersonal interactions and professional networks play a role in shaping individuals’ perspectives on the use of AI in healthcare.
3. Organizational level:
Data protection concerns: The respondent’s concerns about data safety and the use of personal health data by big technology companies reflect organizational factors. The policies and practices within healthcare organizations, as well as external regulations, influence the respondent’s trust in the security and privacy of patient data.
4. Community level:
Policy and regulation: The respondent emphasizes the need for policymakers to address data protection and regulation of AI in healthcare. This highlights the importance of community-level factors, such as government policies and regulations, in shaping the ethical and legal frameworks surrounding AI use.
5. Societal level:
Perceived benefits and risks of AI: The respondent’s perceptions of AI are influenced by broader societal factors, including societal expectations, technological advancements, and media portrayals. The perceived benefits of AI, such as improved diagnosis and early disease detection, align with societal interests in advancing healthcare technology. Conversely, concerns about data privacy and potential negative impacts reflect broader societal debates about the ethical implications of AI.
6. Research and academic level:
Interest in research findings: The respondent expresses interest in the interviewer’s research on AI perspectives among medical practitioners. This demonstrates the importance of research and academic endeavors in generating knowledge and informing discussions around AI adoption in healthcare.
By examining the findings through the socio-ecological lens, we can better understand the multiple levels of influence that shape individuals’ attitudes and perceptions toward AI in healthcare. The model highlights the interconnectedness of individual, interpersonal, organizational, community, societal, and research factors in shaping the acceptance, concerns, and recommendations related to the use of AI in the medical field.

To explore a potential for a higher level of abstraction in qualitative analysis, we then prompted ChatGPT to “Please apply a Marxist lens to your original analysis of the transcript.” In our eyes, the Marxist lens could serve as an example for a more abstract but potentially useful perspective in a real-life investigation of the impact of further technologization of client–provider interactions. The resulting analysis provided by ChatGPT (Box 4) moves beyond the themes initially identified and, in our eyes, proposes a surprisingly nuanced assessment of the data. Several aspects, including, for example, the notions of dehumanization of patients and commodification of health and healthcare, move beyond verbatim content of the interview provided while remaining true to the conversation’s general arguments.

**Box 4.** ChatGPT Investigating the Data From a Marxist Perspective.

Applying a Marxist lens to the original analysis of the transcript involves examining the social and economic dynamics at play and analyzing how power, class, and capitalism influence the perspectives and discussions around AI in healthcare. Here’s an interpretation from a Marxist perspective:
1. Power dynamics and class struggle:
The mention of trust and the desire for a personal connection between doctors and patients highlights the importance of the doctor–patient relationship. From a Marxist perspective, this relationship can be seen as an expression of power dynamics within the capitalist healthcare system. Doctors hold a position of power and authority, while patients are positioned as recipients of care. The introduction of AI in healthcare raises questions about how power dynamics may shift, potentially reducing the agency and autonomy of patients and reinforcing the dominant position of doctors.
2. Capitalist influence on technology:
The mention of big technology companies and their control over personal health data raises concerns about the capitalist influence on AI in healthcare. Marxist analysis would highlight how capitalist interests drive the development and implementation of AI technologies, with companies seeking to monetize data and maximize profits. This raises ethical considerations around the commodification of health information and the potential exploitation of patients’ data for financial gain.
3. Alienation and dehumanization:
The discussion around the limitations of AI in replicating the personal connection and trust that patients have with their doctors points to the issue of alienation and dehumanization within the healthcare system. From a Marxist perspective, the introduction of AI may contribute to the further commodification of healthcare, reducing patients to mere data points and potentially devaluing the human touch and empathy that doctors provide. This raises concerns about the erosion of meaningful human interactions and the potential for healthcare to become more transactional and profit-driven.
4. Class disparities in access and decision-making:
The mention of rare diseases and the potential of AI to support diagnosis in those cases raises questions about how AI may exacerbate existing class disparities in healthcare. Marxist analysis would emphasize how access to AI technologies is often unevenly distributed, with marginalized communities and lower socioeconomic classes facing barriers to entry. This further entrenches existing inequities in healthcare, with privileged individuals having greater access to advanced diagnostic tools and personalized care.
5. Labor implications and job displacement:
The potential for AI to automate certain tasks, such as radiology or surgery, raises concerns about the impact on healthcare professionals’ livelihoods. From a Marxist perspective, this raises questions about job displacement and the exploitation of labor within a capitalist system. The introduction of AI technologies in healthcare may lead to the concentration of power and wealth in the hands of those who control and profit from these technologies, while potentially displacing healthcare workers and exacerbating income inequality.
By applying a Marxist lens, we can uncover the underlying power dynamics, class struggles, and capitalist influences that shape discussions and perspectives around AI in healthcare. This analysis highlights the need to critically examine the social, economic, and ethical implications of AI implementation, ensuring that healthcare systems prioritize human well-being, equity, and the collective good rather than further entrenching capitalist dynamics and class inequalities.

Finally, we wanted to see how ChatGPT would perform when instructed to apply a lens that, in our eyes, would not fit the provided data and identified themes. We used the prompt “Please use the ‘5C psychological antecedents of vaccination’ as proposed by Betsch and colleagues to sort and interpret your previous results when analyzing the transcript,” a request which ChatGPT completed without highlighting the apparent mismatch between interview topic and the originally vaccine-specific 5C model (Betsch et al., 2018) (Box 5). In the resulting description, ChatGPT correctly identified the model and its components and drew several links between components and AI-specific themes in the interview that we ourselves had not considered. Nevertheless, the general application of the 5C model to this dataset seemed forced and suggested ‘overfitting’ of themes to model components. For framework-related prompts, ChatGPT seemed to generally have focused on applying and adapting one theme to each framework component, omitting several previously identified themes in the process.

**Box 5.** ChatGPT Applying the Vaccine-Specific 5C Model to Its Results.

Certainly! The 5C psychological antecedents of vaccination, as proposed by Betsch and colleagues, are a framework that considers various psychological factors influencing vaccination behavior. Applying this framework to the previous results from the transcript analysis, we can sort and interpret the findings as follows:
1. Complacency:
Limited awareness and understanding of AI: The respondent’s limited understanding of AI in healthcare reflects a certain level of complacency, indicating a lack of perceived urgency or need for AI integration in their practice. This suggests that raising awareness and highlighting the potential benefits of AI may be necessary to address complacency.
2. Confidence:
Varied attitudes toward AI among medical professionals: The respondent’s mention of colleagues expressing different attitudes toward AI indicates a lack of consensus or confidence in its use. Some colleagues may be more confident and open to adopting AI, while others may express skepticism or resistance. Building confidence in AI technologies through training, evidence-based information, and successful case studies could help address this variation in attitudes.
3. Constraints:
Trust and privacy concerns: The respondent’s concerns about data safety and the use of personal health data by big technology companies reflect constraints related to trust and privacy. These concerns may act as barriers to embracing AI in healthcare, as individuals may be hesitant to share sensitive information or rely on technology they perceive as a potential threat to privacy. Addressing these constraints is crucial in fostering acceptance of AI.
4. Calculation:
Balancing benefits and risks: The respondent’s mention of both potential benefits (e.g., improved diagnosis and early disease detection) and risks (e.g., data breaches and loss of doctor–patient relationship) indicates a process of calculating the pros and cons of adopting AI. This suggests that individuals weigh the potential advantages of AI in healthcare against perceived risks, emphasizing the need for comprehensive information and clear communication of the benefits while addressing concerns.
5. Collective responsibility:
Policy and regulation: The respondent’s emphasis on the role of policymakers in ensuring data protection and regulating AI reflects the importance of collective responsibility. Addressing the ethical and legal implications of AI use in healthcare requires collaborative efforts involving policymakers, healthcare organizations, and the broader society to establish guidelines, standards, and accountability measures.
By examining the findings through the lens of the 5C psychological antecedents of vaccination, we can identify the psychological factors that influence individuals’ acceptance and concerns regarding AI in healthcare. The framework highlights the need to address complacency, build confidence, alleviate constraints, facilitate informed calculation of benefits and risks, and foster a sense of collective responsibility. Considering these factors can contribute to a more comprehensive understanding of individuals’ perspectives and inform strategies for promoting the responsible and effective integration of AI in medical practice.

## Discussion

When asked to perform qualitative data analysis following various approaches, ChatGPT was able to produce thematic insights that to a considerable degree aligned with or resembled those produced by an experienced human researcher. In general, we ourselves were surprised by the extent to which utilizing ChatGPT for qualitative data analysis yielded data-driven results. A majority of identified themes remained on a descriptive level with few instances of more interpretative engagement with the data. This is in line with previous observations that ChatGPT performs better in recreating descriptive themes (Morgan, 2023) and that (non-literal) language interpretation or creativity poses a challenge for ChatGPT and similar applications (Ray, 2023).

### Chances and Challenges of ChatGPT-Assisted Qualitative Research

Based on our experiences, we see ChatGPT as potentially impacting qualitative research and analysis. A number of authors have highlighted possible benefits of and challenges when integrating ChatGPT into research education and practice [see Sallam (2023) for a systematic review in the context of healthcare research and practice]. Proposed potential use cases include drawing on ChatGPT when exploring more personalized teaching approaches (Gilson et al., 2023; O’Connor & ChatGpt, 2023), when reviewing literature and obtaining preliminary guidance (Lund & Wang, 2023; Marchandot et al., 2023), when analyzing large datasets or requiring timely results (Sallam, 2023), or when writing and revising academic articles, particularly for researchers with a first language other than English (Marchandot et al., 2023; Sallam, 2023). Regarding qualitative research specifically, authors have recently outlined ChatGPT’s potential to support data analysis, especially for identifying descriptive themes (Morgan, 2023), when combined with clear prompting guidance (Zhang et al., 2023) or when used as an augmentative tool instead of a standalone analysis (Jalali & Akhavan, 2024). We add to this ongoing discourse by presenting comparisons of outputs across several analytic approaches and by comparing ChatGPT’s outputs to those of an experienced human researcher. Table 3 summarizes our observations and the opportunities for future in-depth exploration.

**Table 3.** ChatGPT in Qualitative Research—Observations and Future Opportunities.

Our Observations	Opportunities for Future Examination and Application
⁃ ChatGPT offered step-by-step guidance and examples for applying specific analytic approaches.	**Teaching qualitative research** ⁃ ChatGPT could allow students to capture a sense of analytic approaches. Supervision by experienced human researcher(s) would be required to ensure reliability of ChatGPT outputs. ⁃ ChatGPT could reduce or obviate the insight of assignments meant to gauge student abilities related to descriptive analysis.
⁃ Without being prompted to do so, ChatGPT continued the first parts of the transcript with newly generated content with considerable face validity, assuming the roles of both the interviewer and the interviewee.	**Interview guide development and probing** ⁃ ChatGPT could allow for additional piloting and interview/probing practice (e.g., instructing it to act as an interviewer or interviewee, based on its demonstrated ability to generate full interview transcripts). ⁃ ChatGPT could also help in the ordering or content of questions, extending beyond researcher biases.
⁃ ChatGPT provided (descriptive) analyses of transcript content (including themes developed and a codebook suggestion) with considerable overlap to the themes developed by a human researcher. ⁃ ChatGPT-based analysis is fast and prompts can be flexibly adapted, allowing for several analytic iterations.	**Systematic debriefings** ⁃ ChatGPT could quickly summarize large amounts of data for debriefing sessions (McMahon & Winch, 2018) (corrected/supervised by research assistants). Especially when combined with automated transcription [e.g., using VINK (Tolle et al., 2024)]. **Analysis of large but qualitative datasets** ⁃ ChatGPT could identify qualitative themes emerging across datasets too large for human researchers to comprehensively analyze (e.g., social media content). **Reflexivity and result triangulation** ⁃ ChatGPT could facilitate analytic iterations and establish consistently identified themes or codebook components. ⁃ ChatGPT could compel research teams to challenge their own biases, thereby enhancing data collection, analysis, and presentation approaches.
⁃ ChatGPT embedded themes into broader theoretical discourses, providing more interpretative engagement with the data but also running the risk of overfitting.	**Result interpretation and presentation** ⁃ ChatGPT might offer support when linking findings to broader theoretical discourses and could spark novel ideas on potential interpretative nuances. However, theoretical engagement requires close supervision and critical investigation regarding theory fit.

### Comparison Across Process Stages and Repeated Analyses

While in our work the human researcher provided insights across several analytic steps (i.e., sharing both codes and themes) without being explicitly asked to do so, ChatGPT only provided themes when prompted to perform the analysis. When specifically prompted to provide a codebook that could be used when thematically coding similar transcripts, ChatGPT was able to do so, but the resulting codebook did not go beyond the themes previously identified. Additionally, when prompted to provide verbatim quotes to support the identified themes, ChatGPT was able to suggest quotes that fit themes in their content but did not consistently highlight when and how it had edited original verbatim text. While these observations highlight areas that would require particular human supervision when using ChatGPT in qualitative analysis, they also link to ongoing discourses on the inherent opacity of generative AI regarding its content generation (Sallam, 2023): ChatGPT provides (often good quality) end results but no insights into the process of achieving these results (e.g., the codebook in qualitative analysis).

Our retrospective attempts to backtrack procedural components to understand ChatGPT’s analytic process (e.g., asking ChatGPT to provide a codebook or verbatim quotes after it had already presented themes) resulted in simulated intermediate steps, which left us with a sense that codes and quotes had been retrofitted onto themes.

Without being directly prompted to do so, ChatGPT presented guidance for conducting Grounded Theory–informed analysis. The output, which was described by ChatGPT as reflecting Charmaz’s approach, in our eyes aligned moreso with Strauss and Corbin (1998) than with the work of Charmaz (2014). Why ChatGPT enacts certain approaches based on prompts or the degree of ChatGPT’s fluency with various scholars are, to the best of our knowledge, not discernable to the user. One potential explanation in the context of our observation may be that ChatGPT is unable to interpret what Charmaz defines as the creation of both data and analysis based on shared experiences between the interviewer, interviewee, and other sources of data (Charmaz, 2014). Therefore, the more systematic approach defined by Straus and Corbin may offer a clearer process for ChatGPT to follow. However, this observation generally highlights how researchers utilizing ChatGPT have to be particularly cautious in contexts where differences within overarching methodologies have to be considered.

When we prompted ChatGPT to perform several analyses in succession within the same conversation, we commonly observed that identified themes overlapped to a much larger extent than when prompting analyses in separate conversations. This observation links to larger discourses on the learning capabilities of ChatGPT and comparable applications: Within one conversation, ChatGPT can ‘learn’ from interactions and adapt approaches according to user feedback and previous prompts; it therefore is plausible that subsequent analyses would be biased by previous results. In cases where several independent analyses are to be performed, we would therefore recommend using separate conversations. When using such separate conversations, there appears to be no indication that results would systematically differ between different researchers (if the exact same prompts are being used) beyond the general variation observed when repeatedly using the same prompt. However, it remains unclear how repeated interactions with many qualitative researchers on a global scale could contribute to further training of ChatGPT. We are not aware of any guidance on how substantive the interaction data needs to be to result in noticeable changes in general response patterns, but if the use of ChatGPT for qualitative research substantially increases, this might result in increased response quality, but also in increased bias if results are influenced by previous interactions. Future research could more systematically explore how ChatGPT’s theme generation might be shaped by previous interactions with researchers on the same or similar data and topics.

### Theory and Rigor in ChatGPT-Assisted Qualitative Research

When prompted to link identified themes to larger theoretical constructs, ChatGPT was generally able to correctly identify framework components and move beyond a descriptive presentation of themes. However, the program also seemed inclined to ‘overfit’ the data to provided theories regardless of their apparent fit. ChatGPT’s precision in identifying themes in the qualitative data and some of its challenges when contextualizing themes within broader social theory (and other lenses through which we make sense of the world such as history and politics) merit discourse on depth and rigor of qualitative data analysis. On the one hand, observing similar themes being identified by both the human researcher and ChatGPT could be considered one marker of rigor in qualitative research, akin to analyst triangulation (Patton, 1999). We therefore encourage future research that explores ChatGPT’s potential as a complement to human analysis; beyond providing themes or codes, ChatGPT, for example, could serve as another resource in processes of triangulation (Patton, 1999) or thematic synthesis (Thomas & Harden, 2008), or in calculations of intercoder reliability (O’Connor & Joffe, 2020).

On the other hand, while acknowledging that the role of theory in qualitative inquiry varies between approaches and disciplinary traditions (Sandelowski, 1993), we follow arguments made by a number of scholars (Anfara & Mertz, 2014; Collins & Stockton, 2018; Emerson et al., 2011; Sandelowski, 1993) that qualitative research is not atheoretical in its nature but that engagement with theory commonly permeates various steps of good qualitative work, from literature searches to writing ethnographic field notes and result presentations. When we prompted ChatGPT to perform one such step, namely, to embed identified themes into broader theoretical discourses, ChatGPT to a limited degree seemed capable to link the identified themes to higher-level constructs if provided with specific instructions (and might perform even better with more data and more elaborate prompting). However, such engagement with theory also must be coined by a critical questioning of fit and appropriateness of the employed lenses, and while it is highly unlikely that any human researcher would have unquestioningly applied a vaccine-specific model to research on AI use in medical practice, ChatGPT did not comment on this apparent mismatch. ChatGPT’s application of the 5C model to the data was interesting in the sense that it sparked new ideas on potential lenses for further investigation (e.g., interpreting the lack of awareness for AI applications in medical practice under the frame of complacency). However, the program’s performance also emphasized the need for accompanying human reflection as a means to gauge framework fit.

### Biases and Reflexivity When Employing ChatGPT

Across analytic approaches, ChatGPT emphasized its own limitations regarding qualitative data analysis. However, we did not see any indication regarding an acknowledgment of these limitations within the identified themes. As qualitative researchers, we would argue that reflexivity, including acknowledgment of and reflections on our own biases, theoretical lenses, and situated identities, should form a key part of our analysis: While we may not be able to identify all our biases—in fact, we certainly are blind to many of them—we are expected to be aware that biases exist and to try to reflect on how these biases affect what we see in the data. To the best of our knowledge, and in line with our interactive experiences with the program, ChatGPT cannot be expected to perform such reflexivity. It could be argued that the enormous breadth of data used for training ChatGPT might bestow it with something akin to history and perspective. However, reflecting on biases inherent to this training data (and the processes of its production) (Ray, 2023) and providing contextual information not inherent to the textual data (e.g., non-verbal cues and other observations during the data collection activity) remain the responsibility of the interacting human. In interrogating the biases presented in ChatGPT outputs, we see opportunity for a more vigorous conversation among thoughtful qualitative researchers about their own relationship to the data, the study processes, and the nature of knowledge co-production.

### Ethical Considerations

Utilizing the full capabilities of ChatGPT in academic research raises ethical concerns (Parray et al., 2023; Sallam, 2023). For this work, we used an interview and transcript specifically created for this purpose, giving us full ownership of the data and exempting us from a need to acquire ethical and data protection approvals. Before we can recommend researchers to expand systematic exploration and utilization to real-world qualitative data, we urge in-depth considerations of data protection principles and ethical foundations. While first applications drawing on ChatGPT declare sufficient data protection, we note a need for institutional review boards and similar bodies to develop clear and comprehensive research guidelines that outline (in)acceptability of using ChatGPT or other platforms when engaging with specific types of data or for certain analytic purposes. In the meantime, we recommend researchers to employ a conservative approach, which would include refraining from loading sensitive datasets for analysis into ChatGPT. We also urge that researchers obtain ethical and legal assessments regarding data protection and confidentiality from institutional review boards and other bodies familiar with the specific context and data types.

### Ongoing Developments Beyond ChatGPT

Generative AI applications, and LLMs in particular, are rapidly evolving, with various applications beyond ChatGPT being available as of this writing. For the purpose of this article, we focused on ChatGPT as the most well-known, freely available, and not qualitative research-specific application. However, certain observations might differ if utilizing a different application (especially applications powered by a different LLM not based on OpenAI technology, such as Google Bard). From a practical perspective, several applications [both freely accessible (e.g., perplexity.ai with a limit to the files per day in the free version) and paid (e.g., GPT-4)] at the time of writing allow for directly uploading files, which would overcome the challenge of splitting individual transcripts to align with prompt character limitations. While we do not expect there to be a systematic difference in the results obtained if the transcript is uploaded in several segments, as we did in our work, or as one single text file, we see this upload function to greatly facilitate the analysis of more than one transcript. Given the ethical challenges regarding data privacy highlighted above, some applications emphasize increased privacy (e.g., perplexity.ai); however, to the best of our knowledge, the question whether these privacy measures from an ethical perspective are sufficient for sensitive respondent data remains unanswered. Finally, a number of companies developing software specifically for qualitative research have started to include AI applications as paid add-ons (e.g., MaxQDA, NVivo, and ATLAS.ti), in many cases powered by OpenAI technology, which include specific functions (e.g., data summaries or AI coding). While the general aims of these functions overlap with what we explored in this article, we expect experiences to vary when utilizing paid, qualitative research-specific applications with pre-defined functions as compared to the less formalized utilization of LLMs directly. We call on researchers working with the various available tools to share their experiences and lessons learned.

### Limitations

There are a number of ways in which gold-standard qualitative studies would go beyond the methodological approaches employed in this article. First, we only used one transcript for our presented assessment. While we acknowledge that obtaining in-depth thematic insights usually would require several transcripts (as also highlighted by ChatGPT in its outputs), using a single transcript allowed us to do side-by-side comparisons of human and ChatGPT engagement with the full dataset; we encourage further research that draws on larger datasets (provided data privacy and ethical regulations can be maintained). Similarly, our decisions regarding the interviewer, interviewee, and topic of the interview, as well as the fact that both the interviewer and the interviewee were aware of the interview objective to assess the analytic potential of ChatGPT, may have introduced biases. These decisions were made with the overarching goal to obtain a freely usable, in-depth transcript on a health- and technology-related topic in a timely manner, and while we therefore would not use the obtained insights to draw conclusions regarding the topic of the interview, we do believe that the transcript provides a useful starting point for exploring the general potential of AI-assisted analysis.

Additionally, while we asked ChatGPT to perform analysis using several approaches, the human researcher performed only one approach due to time and personnel constraints. Throughout our exploration, we aimed to strike a balance across two conflicting priorities: We wanted to minimize bias across the human analysis versus the ChatGPT analysis, yet we also wanted to involve multiple (human) analysts in each approach. The creation of two larger-but-completely-separated teams was not feasible due to time and resource constraints, but we aimed to facilitate analyst triangulation [or the process of bolstering result trustworthiness by involving multiple analysts to co-code data or review findings (Patton, 1999)] via the involvement of other team members (including the interviewee and senior researchers) in debriefings and discussions on codes and themes. The involvement of the interviewee-researcher formed a unique, and in real-life research unlikely, overlap between analyst triangulation (Patton, 1999) and member checking (Thomas, 2017), and while this process in our opinion helped to ensure trustworthiness of findings for the purpose of this article, we would encourage future research on the potential of AI-assisted qualitative analysis in larger projects and research teams.

Finally, we are not professional AI prompt designers and some of our instructions might seem clumsy at best. We note that this reality likely mirrors the use case of many qualitative researchers who lack familiarity with LLMs. Similarly, we used the freely available (and therefore for researchers flexibly accessible) ChatGPT (based on GPT-3.5) for our work. While not freely available, GPT-4 may perform considerably better on some tasks (Kocoń et al., 2023). Despite these limitations, we see our insights as contributing to an open debate on the potentials and drawbacks of LLMs as a qualitative analysis tool, and we encourage more systematic exploration of various tools and prompts.

## Conclusion

When exploring the potential of ChatGPT in qualitative analysis, we were surprised by its apparent ability to provide useful insights across several steps of the analytic journey. While results were particularly convincing for the identification of descriptive themes in the provided data, ChatGPT could also provide more interpretative contributions, for example, when embedding themes into broader theories, as long as the researcher critically assessed applicability and usefulness of the specific theory to avoid overfitting.

Across potential use cases identified in our work, we echo scholars who argue that any use of ChatGPT in research and practice must be accompanied by careful (human) observation and reflection (Doshi et al., 2023; van Manen, 2023). However, given the rapid development of ChatGPT and similar applications, we expect the relevance of such tools to increase in a broad spectrum of fields, including in (qualitative) research. Based on our experiences, ChatGPT might not only be able to provide descriptive summaries of data or support with teaching and skill development as previously suggested, but might also hold potential for supporting or conducting specific analytic steps under human supervision.

Moving forward, we call for qualitative researchers to build on these first insights to further our understanding whether and under what conditions ChatGPT and similar applications can support qualitative work. We specifically suggest three directions for further exploration: First, we aimed to provide insights across several approaches to qualitative inquiry, but further research is needed that systematically assesses ChatGPT’s performance across specific approaches following gold-standards of qualitative research from a spectrum of epistemic communities. We particularly invite scholarship that explores the potential of ChatGPT within and across the four tiers of Crotty’s framework [epistemic, theoretical perspective, methodology, and methods (Crotty, 1998)] to facilitate informed decision-making on potential use cases and drawbacks for future research projects. Second, while ChatGPT in our eyes is the most prominent freely accessible LLM-based application, a number of comparable models exist or are in development, as well as several applications building on these LLMs that are specifically targeted at qualitative research. We would encourage researchers to share experiences across different models and applications to bolster our understanding of tool-specific benefits and drawbacks. Finally, moving from small amounts of data created explicitly for upload into ChatGPT to real-life research poses several ethical and data protection challenges. We therefore call for insights on the legal and ethical dimensions of various use cases of ChatGPT and other novel tools.

With this article, we hope to encourage an open discourse among qualitative scholars to ensure that, as a field, we leverage this new potential where possible without impeding the rigor of qualitative analysis—but that we also are aware of possible pitfalls, such as rapid but decontextualized analysis, that could hamper high-quality teaching and research.

## Supplemental Material

Supplemental Material - Prompts, Pearls, Imperfections: Comparing ChatGPT and a Human Researcher in Qualitative Data Analysis Supplemental Material for Prompts, Pearls, Imperfections: Comparing ChatGPT and a Human Researcher in Qualitative Data Analysis by Jonas Wachinger, Kate Bärnighausen, Louis N. Schäfer, Kerry Scott, and Shannon A. McMahon in Qualitative Health Research

Supplemental Material - Prompts, Pearls, Imperfections: Comparing ChatGPT and a Human Researcher in Qualitative Data Analysis Supplemental Material for Prompts, Pearls, Imperfections: Comparing ChatGPT and a Human Researcher in Qualitative Data Analysis by Jonas Wachinger, Kate Bärnighausen, Louis N. Schäfer, Kerry Scott, and Shannon A. McMahon in Qualitative Health Research

Supplemental Material - Prompts, Pearls, Imperfections: Comparing ChatGPT and a Human Researcher in Qualitative Data Analysis Supplemental Material for Prompts, Pearls, Imperfections: Comparing ChatGPT and a Human Researcher in Qualitative Data Analysis by Jonas Wachinger, Kate Bärnighausen, Louis N. Schäfer, Kerry Scott, and Shannon A. McMahon in Qualitative Health Research

## Note

1. Asking for additional analysis using different approaches in the same conversation sometimes led to ChatGPT returning the same themes across analytic approaches. To ensure independent computing of the analysis, we therefore decided to use separate conversations for each approach.

## References

[bibr2-10497323241244669] Anfara V. A. Mertz N. T. (2014). Theoretical frameworks in qualitative research. Sage Publications.

[bibr3-10497323241244669] Betsch C. Schmid P. Heinemeier D. Korn L. Holtmann C. Böhm R. (2018). Beyond confidence: Development of a measure assessing the 5C psychological antecedents of vaccination. PLoS One, 13(12), Article e0208601. 10.1371/journal.pone.0208601 30532274 PMC6285469

[bibr4-10497323241244669] Braun V. Clarke V. (2019). Reflecting on reflexive thematic analysis. Qualitative Research in Sport, Exercise and Health, 11(4), 589–597. 10.1080/2159676x.2019.1628806

[bibr5-10497323241244669] Charmaz K. (2008). Grounded theory as an emergent method. In S. Nagy Hesse-Biber & P. Leavy (Eds.), Handbook of Emergent Methods (pp. 155–172). The Guilford Press.

[bibr6-10497323241244669] Charmaz K. (2014). Constructing grounded theory. Sage Publications.

[bibr7-10497323241244669] Collins C. S. Stockton C. M. (2018). The central role of theory in qualitative research. International Journal of Qualitative Methods, 17(1), Article 160940691879747. 10.1177/1609406918797475

[bibr8-10497323241244669] Crotty M. J. (1998). The foundations of social research: Meaning and perspective in the research process. Sage Publications.

[bibr9-10497323241244669] Doshi R. H. Bajaj S. S. Krumholz H. M. (2023). ChatGPT: Temptations of progress. The American Journal of Bioethics, 23(4), 6–8. 10.1080/15265161.2023.2180110 36853242

[bibr10-10497323241244669] Emerson R. M. Fretz R. I. Shaw L. L. (2011). Writing ethnographic fieldnotes. University of Chicago press.

[bibr11-10497323241244669] Feuston J. L. Brubaker J. R. (2021). Putting tools in their place: The role of time and perspective in human-AI collaboration for qualitative analysis. Proceedings of the ACM on Human-Computer Interaction, 5(CSCW2), 1–25. 10.1145/347985636644216

[bibr12-10497323241244669] Gilson A. Safranek C. W. Huang T. Socrates V. Chi L. Taylor R. A. Chartash D. (2023). How does ChatGPT perform on the United States Medical Licensing Examination (USMLE)? The implications of large language models for medical education and knowledge assessment. JMIR Medical Education, 9, Article e45312. 10.2196/45312 36753318 PMC9947764

[bibr13-10497323241244669] Graneheim U. H. Lundman B. (2004). Qualitative content analysis in nursing research: Concepts, procedures and measures to achieve trustworthiness. Nurse Education Today, 24(2), 105–112. 10.1016/j.nedt.2003.10.001 14769454

[bibr14-10497323241244669] Jalali M. S. Akhavan A. (2024). Integrating AI language models in qualitative research: Replicating interview data analysis with ChatGPT. SSRN Electronic Journal. 10.2139/ssrn.4714998

[bibr15-10497323241244669] Jiang J. A. Wade K. Fiesler C. Brubaker J. R. (2021). Supporting serendipity: Opportunities and challenges for human-AI collaboration in qualitative analysis. Proceedings of the ACM on Human-Computer Interaction, 5(CSCW1), 1–23. 10.1145/344916836644216

[bibr16-10497323241244669] Kocoń J. Cichecki I. Kaszyca O. Kochanek M. Szydło D. Baran J. Bielaniewicz J. Gruza M. Janz A. Kanclerz K. Kocoń A. Koptyra B. Mieleszczenko-Kowszewicz W. Miłkowski P. Oleksy M. Piasecki M. Radliński Ł. Wojtasik K. Woźniak S. Kazienko P. (2023). ChatGPT: Jack of all trades, master of none. Information Fusion, 99, Article 101861. 10.1016/j.inffus.2023.101861

[bibr17-10497323241244669] Lumivero . (2023). Revolutionizing text data analysis with AI autocoding with NVivo. https://lumivero.com/resources/blog/revolutionizing-text-data-analysis-with-ai-autocoding-with-nvivo/?utm_source=twitter&utm_medium=social_organic&utm_campaign=blog&utm_id=engagement

[bibr18-10497323241244669] Lund B. D. Wang T. (2023). Chatting about ChatGPT: How may AI and GPT impact academia and libraries? Library Hi Tech News, 40(3), 26–29. 10.1108/LHTN-01-2023-0009

[bibr20-10497323241244669] Marchandot B. Matsushita K. Carmona A. Trimaille A. Morel O. (2023). ChatGPT: The next frontier in academic writing for cardiologists or a pandora’s box of ethical dilemmas. European Heart Journal Open, 3(2), Article oead007. 10.1093/ehjopen/oead007 36915398 PMC10006694

[bibr21-10497323241244669] MaxQDA . (2024). AI Assist: Introducing AI to qualitative data analysis. https://www.maxqda.com/products/ai-assist

[bibr22-10497323241244669] McMahon S. A. Winch P. J. (2018). Systematic debriefing after qualitative encounters: An essential analysis step in applied qualitative research. BMJ Global Health, 3(5), e000837. 10.1136/bmjgh-2018-000837 PMC6135453 30233833

[bibr23-10497323241244669] Morgan D. L. (2023). Exploring the use of artificial intelligence for qualitative data analysis: The case of ChatGPT. International Journal of Qualitative Methods, 22, Article 16094069231211248. 10.1177/16094069231211248

[bibr24-10497323241244669] O’Connor C. Joffe H. (2020). Intercoder reliability in qualitative research: Debates and practical guidelines. International Journal of Qualitative Methods, 19, Article 160940691989922. 10.1177/1609406919899220

[bibr25-10497323241244669] O’Connor S. ChatGpt . (2023). Open artificial intelligence platforms in nursing education: Tools for academic progress or abuse? Nurse Education in Practice, 66, Article 103537. 10.1016/j.nepr.2022.103537 36549229

[bibr26-10497323241244669] Owens B. (2023). How Nature readers are using ChatGPT. Nature, 615(7950), 20. 10.1038/d41586-023-00500-8 36807343

[bibr27-10497323241244669] Parray A. A. Inam Z. M. Ramonfaur D. Haider S. S. Mistry S. K. Pandya A. K. (2023). ChatGPT and global public health: Applications, challenges, ethical considerations and mitigation strategies. Global Transitions, 5(1), 50–54. 10.1016/j.glt.2023.05.001

[bibr28-10497323241244669] Patton M. Q. (1999). Enhancing the quality and credibility of qualitative analysis. Health Services Research, 34(5 Pt 2), 1189–1208. 10591279 PMC1089059

[bibr29-10497323241244669] Pope C. Ziebland S. Mays N. (2000). Qualitative research in health care. Analysing qualitative data. BMJ (Clinical Research Ed.), 320(7227), 114–116. 10.1136/bmj.320.7227.114 PMC1117368 10625273

[bibr30-10497323241244669] Rahman M. Terano H. J. R. Rahman N. Salamzadeh A. Rahaman S. (2023). ChatGPT and academic research: A review and recommendations based on practical examples. Journal of Education, Management and Development Studies, 3(1), 1–12. 10.52631/jemds.v3i1.175

[bibr31-10497323241244669] Ray P. P. (2023). ChatGPT: A comprehensive review on background, applications, key challenges, bias, ethics, limitations and future scope. Internet of Things and Cyber-Physical Systems, 3(1), 121–154. 10.1016/j.iotcps.2023.04.003

[bibr32-10497323241244669] Saldaña J. (2011). Fundamentals of qualitative research. Oxford University Press.

[bibr33-10497323241244669] Sallam M. (2023). ChatGPT utility in healthcare education, research, and practice: Systematic review on the promising perspectives and valid concerns. Healthcare, 11(6), Article 887. 10.3390/healthcare11060887 36981544 PMC10048148

[bibr34-10497323241244669] Sandelowski M. (1993). Theory unmasked: The uses and guises of theory in qualitative research. Research in Nursing & Health, 16(3), 213–218. 10.1002/nur.4770160308 8497673

[bibr35-10497323241244669] Strauss A. Corbin J. (1998). Basics of qualitative research: Techniques and procedures for developing grounded theory. Sage Publicaitons.

[bibr37-10497323241244669] Thomas D. R. (2017). Feedback from research participants: Are member checks useful in qualitative research? Qualitative Research in Psychology, 14(1), 23–41. 10.1080/14780887.2016.1219435

[bibr38-10497323241244669] Thomas J. Harden A. (2008). Methods for the thematic synthesis of qualitative research in systematic reviews. BMC Medical Research Methodology, 8(1), 1–10. 10.1186/1471-2288-8-45 18616818 PMC2478656

[bibr39-10497323241244669] Tolle H. Castro M. d. M. Wachinger J. Putri A. Z. Kempf D. Denkinger C. M. McMahon S. A. (2024). From voice to ink (Vink): Development and assessment of an automated, free-of-charge transcription tool. BMC Research Notes, 17, Article 95. 10.1186/s13104-024-06749-0 38553773 PMC10981346

[bibr40-10497323241244669] Tracy S. J. (2010). Qualitative quality: Eight “big-tent” criteria for excellent qualitative research. Qualitative Inquiry, 16(10), 837–851. 10.1177/1077800410383121

[bibr41-10497323241244669] van Manen M. (2023). What does ChatGPT mean for qualitative health research? Qualitative Health Research, 33(13), 1135–1139. 10.1177/10497323231210816 37897694

[bibr42-10497323241244669] Zhang H. Wu C. Xie J. Lyu Y. Cai J. Carroll J. M. (2023). Redefining qualitative analysis in the AI era: Utilizing ChatGPT for efficient thematic analysis. arXiv preprint, 2309.10771. 10.48550/arXiv.2309.10771

