# Supplementary material for: Prompts, Pearls, Imperfections: Comparing ChatGPT and a Human Researcher in Qualitative Data Analysis
Source: Qual Health Res. 2024 May 22;35(9):951–66. doi: 10.1177/10497323241244669 (PMC12202826; doi:10.1177/10497323241244669)
Supplement: Supplemental Material - Prompts, Pearls, Imperfections: Comparing ChatGPT and a Human Researcher in Qualitative Data Analysis [file sj-pdf-1-qhr-10.1177_10497323241244669.pdf]

Prompts, pearls, imperfections; comparing ChatGPT and a human researcher in qualitative data analysis.

Supplemental file 1:

- Full interview transcript (p.1-16)
- Original interview guide (p.17)
- Upload prompts based on *ChatGPT splitter* (p.18-28)

Full interview transcript

I: Hi, ahem, yeah thank you again for participating in today's interview. Ahem, maybe to start off, could you please just tell me a little bit about your, your own day-to-day clinical work?

R: Ok, Yeah, ahem, thanks a lot for having me. Ahem … Usually, ahem our, or my doctor's office is opened from Monday to Friday and it's almost every day, ahem, the same. Usually, in the mornings, ahem we have a patient consultation, ahem, some, ahem have chronic diseases, some have something … more urgent. Ahem, and, yea different sorts of patients, ahem, different backgrounds, younger people, older people, ahem … yes from the area here. So, also many patients that, ahem, I know for a longer time now, … like for … a few years after I started. Ahem, yes, in the afternoon, on some days, we have it as well. We also do some, ahem, visits at patients' homes … and, ahem, we also do some examinations, as for example, ultrasound … examinations that are planned in the afternoon with patients that, for example, come for regular checks, check-ups.

I: Okay, okay, thank you so much. Just … just for me to understand, ahem, is it like in, in your practice, is it just you or are you several doctors sharing one office, so to say?

R: Yea, we are several doctors, ahem. I'm still, ahem doing my specialization, so I also have a supervisor with whom I'm meeting on a regular basis, but who I can also consult if I'm not sure what's the case. Ahem, but yeah, we are like five … five medical doctors working together in the practice.

I: Okay, and all of you have a, ahem … the specialization in, like, just as a GP? Or do you have diverse backgrounds?

R: Yeah, ahem, most, ahem, one has also a specialization in internal medicine, but it's like working more or less as a general practitioner … and, ahem, some have also some, ahem, additional specializations as … emergency medicine, ahem … or something.

I: Okay, okay. So…

R: Generally, we are all working as general, … or all in this office are working as general practitioners.

I: Okay, okay, thank you. And, ahem, that means, so if I understood correctly, like in … in general, you have this, like, mixture of patients throughout the week, sometimes in the

afternoons you have more … specialized, ahem, like diagnostic procedures planned, ahem, things like that. And then, … ahem, but do you also perform surgeries and these kinds of more complicated procedures or is that usually something where you refer your patients to other?

R: It's something we refer … refer patients for, ahem, to … to other clinics or offices, yes.

I: Okay, okay.

R: When … Something that needs the expertise of another specialty.

I: Okay, okay, thank you. Great. Ahem, and your, your patient base, so you said you have basically the full spectrum from rather young patients to rather old patients and from chronic complicated cases and also more day to day, like, smaller emergencies, so to say.

R: Yeah, exactly. There are some patients that have been coming to this office for … 10 or 20 years now. Of course, I'm not there for this long time, but … all the colleagues know … know them quite well and I also had a chance to, to get to know … several of them during the last … two, three years. Ahem, but yeah, for the day to day work, people having a cold or something similar ... Yeah.

I: Ah that's really nice to have this time to really form a relationship with the patients, like basically over, yes, over many years. Ahem, that sounds really nice.

R: [laughs]

I: [laughs]. Thank you. Ahem … So maybe to, to … ahem, move on to the next questions. Ahem, in general, what is your perspective or what do you think about digital technologies in clinical work or in clinical practice?

R: I think it depends. I think there are many new tools that … ahem, can be used in … ahem, or that could work, make easier or more efficient. Ahem, for example, … ahem, digital, ahem, like we have like this … system in our, ahem office where we can … where we have all the patient's data, all ahem … information we put into it, all examinations that were done. We could check that. I think there's also like, ahem … there are more things that could be done, but I'm not yet, especially Germany, ahem, where it always takes a long time until technology is adopted [laughs]. … Ahem, so yeah, I think there are things that can … make work easier, but you also depend on it. So if one day this … system we use here in the office is not working, ahem, that makes things difficult [laughs] because everything, ahem, is based on that. And … ahem … but yeah, I think there are chances, but there are also, like … challenges, ahem, in clinical practice.

I: Okay, yes. So, if I understood it correctly, there, like, the way it's currently implemented at your own practice, ahem, you mainly use it for, like, record keeping and for, ahem, basically, yea, structuring your records. Ahem, and there are certain things if I understood it correctly, where you would say, … well, you would like to do, or you would be open to implement it more strongly, but there are certain ways in which maybe regulations are like on a larger, … like you said on a national level, certain things might not yet be possible?

R: Yeah, especially connecting … like, for example, ahem, office with hospitals, that we can send information to each other more easily and … yeah. Stuff like that.

I: Ah Okay. Okay. … Okay.

R: And another way I have been in contact with that, it's like also some patients having those watches, those smart watches.

I: Hmm, okay.

R: Some also like … ahem, checking for example your, your … ahem, yea some basic information on … like your heartbeat or something like that. Ahem … I don't know how that exactly works, honestly. I should, but [laughs] … maybe I should teach myself more about that. But yeah, I know that some people are tracking … are tracking that, ahem, using this. And I've also read about cases … cases, ahem, where … where those watches, ahem … Warn patients about like, say, ahem rhythm anomalies, but yeah. … But I never had that case, ahem … seen. Like, I've never seen that myself so far.

I: Okay. Okay. But, ahem, were there instances where … you already have used data from, from smart watches in your own interactions with patients?

R: No, no, I've never used that so far.

I: Okay.

R: Like I have some patients who talked … about that, bit for them them, they were healthy. So, ahem … in my view. So, there was nothing, ahem, yeah … like no abnormal, ahem, like, yeah.

I: Okay. Okay.

R: Nothing bad [laughs].

I: Okay. So that data, in a way they have it available through their … watches or whatever, ahem, but it doesn't yet play any role in your … in your day to day work, so to say? They mentioned about it, but it's not integrated?

R: Not yet. Not yet [laughs].

I: Okay. Okay.

R: It's interesting. I think it's super, it's interesting, but yeah. … I haven't had the chance to … to learn how to make use of it, … but I'm interested in to it. So …

I: Yeah. Okay. And also, your other colleagues, like … especially the colleagues within your office … like they are also not yet working with things like that?

R: Hmm … not, not people I have direct contact to, like we had some discussions about it. I also heard about some applications in … in clinical work, but not, ahem, in my, in my close environment.

I: Okay. Okay. Thank you. … Maybe moving on to this broader topic of, ahem, artificial intelligence.

R: Hmmm:

I: Ahem, what is your impression of that and … what have you heard about, yeah, this buzzword of artificial intelligence, so to say?

R: In general, or, ahem, only like in the field of medicine?

I: Ahem, I would say now to start off in general, and then we can move on to the clinical context.

R: Okay. … In general, it feels like, ahem … everybody's talking about ChatGPT [laughs].

I: Hmm

R: But … yeah, I … I have to say, ahem, I'm not sure about my feelings on it. I think it can help a lot, but … I think, like, when it gets possible to … to, ahem, to fake interviews, things that people have said … fake videos, maybe also fake scientific data, … it makes … Yea, I'm a little afraid that this might make things complicated or … even, like … could be used by … I don't know … political actors or some, something like that to, for … many, many things that might not be … might have, ahem very negative impact. So yeah, that's something I don't know so much about, but … I'm a little afraid of that those … those …. technical tools one can use them in a quite bad way [laughs].

I: Yeah. Okay. Okay.

R: But yeah, generally I think they're also, like, positive perspectives.

I: Okay. Okay. Okay. And …. So, this, these concerns that you described, they … basically apply to the general, … like, to everyday life, so to say, or all aspects of life? Or are there specific aspects that you would apply them to?

R: I would say, ahem, to all aspects of life. Like, especially I, I have been thinking about it like in this … about this, in this political field, but … I think, yeah, they're also like, … I think we will have, at some point, it will be possible to have artificial intelligence … in so many different … ahem, yea, facets of your life. So … yeah.

I: Okay. Okay.

R: Also, medicine. Yeah [laughs].

I: Yeah. Ahem, yes, that would have been [laughs] that would have been my next question. So, the … is there already, like, have you already heard about it in the context of clinical work or of medicine?

R: There are like two things I've heard of. One … are, ahem, softwares helping, for example, analyzing … ahem, basically data from examinations, like for example, ECG or, ahem … ahem, or, yeah, other, like similar, similar things, analyzing x-rays or … ahem … yeah, other, ahem ... Other, other imaging techniques. Ahem, …. yeah. That's like the two things I've basically heard of … that there are studies are, or, also already in institutions using those technologies who are getting, … who were in some cases better than human, ahem, human, ahem, doctors in analyzing. Yeah.

I: Okay. Wow. Okay.

R: For Imaging processes.

I: And then analyzing, so to say, to reach to … to a more specific diagnosis or, or more specific recommendations than a human would in this context?

R: I'm honestly not sure. It's quite some time ago, but I think it's, it’s less recommendations, but more, like, diagnosis. Ahem, or for example, locating something, or like … seeing small

ECG changes that … maybe can be overlooked by, by … by other, like, people, ahem [inaudible].

I: Okay. Okay. Yeah. It's really interesting. Yeah.

R: But I’m no, I'm not an expert on that. So … [laughs]

I: Yes. So, you said, sorry, if I … I didn't catch it, you said there are two, two sort of fields that you already, ahem, heard about it. So, one being this, ahem … broader, like, analysis of examination data? And what was the second one? Sorry.

R: Yeah. That were decision support system, I think it's called decision support system.

I: Ok.

R: I'm not 100% sure, but yeah, ahem, it's like, ahem, it's also used for diagnosis. And … like, I think there are also already, like, apps on that, that are working with artificial intelligence, ahem, for example. And it's also tested in the clinical field where you put, like, the symptoms.

I: Ah.

R: And, and I think also, like, data from examinations as, ahem, yeah …

I: Mhm.

R: As, for example, yeah. Images… or… blood values into…

I: Okay.

R: That might form like… chances of different diagnosis, maybe finding something that, aehm, we wouldn't have thought about.

I: Mhm.

R: Yeah, and I think that exists, so far exists, in, like, this field where it's tried out and also already like… apps for…for smartphones that everybody can use. Where you can, like, check your symptoms.

I: Okay.

R: Yeah. Not sure how that exactly works. And… like…

I: [laughs] Okay. Okay. Yeah, and have you, in any way, already used any of these… like, or artificial intelligence in any form in your work?

R: Ehm, not in my work. I tried out one of these apps [laughs] once, aehm, but that's quite some time ago.

I: Okay.

R: Yeah.

I: The diagnostics, like where you put symptoms and things like that…

R: Ehm, and quite some time ago, I don't remember…remember too much. I thought, oh yeah, that's interesting. But then I didn't follow up.

I: Okay. And what was your experience with it or what did it say so to say? [laughs]

R: As mentioned it was already some… some time ago. Ehm, I'm not sure, like… like it showed like five diagnosis rates that, okay, yeah, that might be possible… [both laugh] Okay. I just put that. Ehm, yeah and compared to when you Google your symptoms, it doesn't say like, oh yeah, you might have this very bad disease and this and this, but it's like also working a little more.

I: Aah.

R: I think that's really real probabilities and nothing where a tumour that you might have… just when you only have headache or something.

I: Okay.

R: So, ehm…

I: So, the results are less drastic or like unlikely drastic.

R: Yeah. Yeah. But I think, I…, it also showed them. So that's possible… You should maybe go and check, but ehm yeah, don't like… it didn't make you as much… or like, I felt that when I would read it both, it wouldn't make me like panic as I would just Google it and be like, oh my God.

I: Okay. Okay. Is that something?... [both talk at the same time] Sorry…

R: It's quite some, it was quite some time ago and I was still studying and yeah. Ehm. And so, I don't remember too much, but that's what I, but I think that I remember quite some time ago.

I: Okay. Is that something that already comes up in your interactions with your patients? Like, do people come to you and talk about, aehm, having used AI in any form or expecting you to use it in any way?

R: Ehm… No, not yet. People who Google, this patients who Google their symptoms or use Google or some other search engine, ehm, for looking up their symptoms, yes, that happens, but, aehm, I… don't remember that anybody that would… using AI or some, at least they didn't talk some… or… and nobody expected to use it.

I: Okay. Okay.

R: I once had a discussion with a patient who asked if… if we know about… like who just read some newspaper article about that. Aehm. That was that.

I: Okay. And, but that patient also was more just interested in hearing your opinion about it?

R: Yeah. Yeah. Yeah. So, no… no… no expectations yet. I think most of it is still on file.

I: Okay. Okay. Okay. But based on your experience, aehm, the, like it might actually be less concerning if people would put their symptoms into… these kinds of AI systems, then just Googling it and, and come to you being concerned of having a very severe disease?

R: I think [laughs] it's… I think if you have symptoms and go for a check-up, it's always good… [both laugh] Yeah. It might be good that they're not that concerned, but it would be good if like, aehm, anyways. Aeeehm… But I… I'm not sure about this. Like… like this is one… one like point or one layer, but I also… think that I shouldn't make people think or that the fact that they, people, should not think… the fact that they have checked their like symptoms or something else or feeling with an AI that, that yeah… that they don't have to go

to like some medical practitioner to check it. Aehm, so… aehm, that's it. Oh yeah. Aehm, I… I only use that app now. It replaces all my apps.

I: Ah okay…

R: Because I think that medical practice are like… are like, yeah, our job is more than just like have a list of symptoms and there's also like a person behind it. And you see that person, aehm, how the person presents themself, aehm, how he or she behaves, how… yeah, you might also think it's just like, how do you feel? And that's… that's stuff that I think that's difficult to be replaced by an AI. Aehm… So… I think that like… aehm, only talking about diagnosis now, not about… [laughs] about recommendations or treatment. I think that's something completely different, where it's, aehm, like, where I'm not sure how we could use AI there or what could be there because they, I think they really need that, aehm… like a relationship somehow, especially with chronically ill patients. Aehm… They need something, somebody to trust. And like, it's trust always goes first. So yeah. Aehm… So, I'm not sure how it would work with that. But I think, aeehm, we were still at diagnosis, so... And also, like psychological, psycho… aehm, is psychosomatic, also the word in English?

I: Mhm.

R: Aehm… Yeah. Symptoms of… and their connections with aehm, with the body that I think, I'm not sure how this can be caught by an AI. Maybe it can. I… Aehm… And also, patients like describe or go share, aehm, symptoms maybe differently. They might have the same disease, but for one person… okay. People have different symptoms, but people also like say, describe… aehm… for example, a pain differently. I think therefore it's, I'm not sure if you can just put it on a scale on AI and say, okay, yeah, that's, that works perfectly. You don't need a doctor here anymore or somebody else…

I: Okay. Okay. Okay. So, in that sense, would you say that… you see… like you will have experienced or you have heard about it and you could also imagine using it in the context of like as diagnostic support, so to say?

R: Yeah, I think like that's the point. Like I wouldn't say that it kept like replace like… aehm… diagnosis… and like, aehm, you're really talking to people, but it could support it. Maybe when you can enter symptoms there and maybe it says, oh yeah. When you don't know what that could be. And maybe I heard of it, like I heard that those systems are simply used at aehm… ambulan… aehm… clinics for rare diseases.

I: Mhm.

R: So, maybe it finds something that you haven't been considering because maybe you just didn't know… because there's like so many rare diseases. I'm not sure if anybody could know all of them… with… aehm… symptoms and recommendations. Aehm, so, I think therefore it could really be helpful as well, but… I think it couldn't replace. [laughs]

I: Yeah. Yeah. Okay.

R: …consultation, but it could support. And I think that's really… therefore… I found that super interesting.

I: Mhm. Mhm. Yeah, no, definitely.

R: Or maybe saying, oh, that doesn't fit here. Maybe it's like… aehm… when you're saying you, so you can have two diseases at the same time…. Aehm… Oh yeah. But I'm not worrying about both…

I: Okay. So, you described that like one of the… the key opportunities related to AI now in your work, you would see in this like diagnostic support or like… maybe identifying certain patterns that might be… that otherwise might be overlooked. If I understood correctly…?

R: I would… Yeah. Yeah. That's what I would say.

I: Okay.

R: For the diagnostics. [laughs]

I: Mhm. Are there any other like opportunities, aehm, you would see? Like now or in future also looking at how the… like potential developments in the field?

R: Like… for treatment… I'm not so sure on that. I think maybe it can look for studies, recommendations... Aehm… There are the official recommendations for several diseases. Maybe it could help, especially in the case of rare diseases at some point. But also in this case, I think it doesn't… repl… it's not really possible to replace a physician there or other medical practitioners. Like… That's also the case. As I mentioned before, you need connection to the person. Or at least I would say that you need that. Aehm… A person who like doesn't see you like as members, but as the person you are, knows your social environment… And that's also something super important and… I'm not sure how like… the intelligence should deal with that…

I: yeah...

R: ehm… Also, we are living in an age of shared decision making. I'm not sure how… I could try to make decisions with a patient together and support the patient in making decisions. How that would work out without that personal connection. And also… like after making a decision, you're doing checks on your patient. How… aehm… maybe also how this person is like maybe changing in her feelings or like in her presentation or behavior. I'm not sure if a… AI could grasp that when you have been seeing a patient for… over years. And see how they are behaving, how they are talking, how they are maybe feeling…

I: Mhm.

R: I think it wouldn't be possible to replace that… Aehm, and also like you can… maybe that would be something like interaction of medication or something like that. Something I could see that, but also like isn't just describing the medication. It's also communicating that it's… how it's important to take it in a certain way. Because I think there are studies on it that a lot of patients, almost half, doesn't take their, aehm, medication as prescribed. Aehm… As I would myself. Yeah. [Both laugh] But also if I'm sick, I'm also not taking everything as I should.

I: Mhmmmm.

R: So, I'm also lying to this 50% that doesn't take it. [both laugh] I think it's important to communicate why and how and where you could maybe leave out once. Aehm, also like forwarding the advantages and disadvantages of medication. Aehm… or like of a combination of medications. Maybe that's the part where I could support too… But… aehm… coming to

side effects and how a patient… aehm… experiences side effects of medication? I'm not sure if things like that are possible to grasp by an AI,, but… but, yeah. Maybe it could like help on this. Okay, yeah… this medication might have these side effects. And then also then we're coming back to diagnostics.

I: Mhm. [laughs]

R: It's connected. [laughs]

I: Okay, okay. So, you mentioned that you as a practitioner who potentially has interacted with the same patient over years and of course has collected all this information and built this relationship over time. Do you think there that potentially an AI if it would also have sort of the same sort of basis of information? Like to imagine that the AI has exactly the same number of interactions or the number of varying descriptions of their symptoms and things like that. So, do you think with having a bigger database for an AI, this could also make it more competent in interacting with the patient? Or would you say, there's just always something that this kind of algorithm or AI could not grasp, no matter how much data it has?

R: A really good question indeed. I'm not sure what AI can do or what it will be able to do in 10, 20 years. So maybe, it's possible if you have cameras everywhere that the AI can analyze a patient's behavior, their face, how they're talking. I'm sure that that will be possible at some point. Another question is if people want that.

I: Okay.

R: So, I think a trustful relationship between patient and health practitioner is important for both. As a patient, I'm also happy when I can go to another - when I have something, and I can go to a doctor with a specialty maybe. Or, also not just be treating myself, but when I can go there and have a trustful conversation. And I know that that's only that person listening to me and no computer that analyzes everything. And that person that maybe tries to understand me and see me and not only to analyze. So, I think, I wouldn't want that as a patient.

I: Mhhh

R: I wouldn't want that as a practitioner, but... yeah…

I: Yeah, this trusted relationship, it's a really relevant point, of course. Could you imagine cases where you yourself would have maybe more trust into an AI in a clinical setting than in a human doctor?

R: I think it always depends on the human doctor [laughs]. I could imagine that in some cases where I'm not really sure about symptoms and explanations I have, how that fits together and there are some things that are a little[...] Maybe they're strange. […] I would say, okay, maybe I'll see AI. Maybe it's something that doesn't appear so often. Maybe some rare side effects – it doesn't have to be a rare disease, but some rare side effects of a standard medication. That happens. I think in those cases, yeah, I think there are situations where I would say: 'Okay, maybe let's check'. Maybe not replacing the doctor, but like, working together.

I: Okay, okay: And how do you think your patients would react if you would integrate, for example, for the diagnostic component, integrate AI into your interactions with them or into your consultations?

R: Ehm, I think [laughs] there would be mixed reactions.

I: Okay.

R: I think there would be some patients that would say: ‘Oh, cool, he's checking up with that AI. Maybe also to not overlook something.’ Maybe there are some other patients who would say: ‘Okay, what are you doing? Do you have to google and look up everything?’ in the interaction.

I: Okay.

R: So, I think there would be mixed reactions. I think that also not at this point, not everybody knows about it. There's not so much knowledge. There's not so much known about how these technologies work. Ehm, so, yeah, I'm not sure about, I think there would be mixed reactions.

I: Okay.

R: But I think when I would like install cameras everywhere and say: ‘Oh yeah, that's just eye tracking, you have to talk with it. I'm just sitting here`.

I&R: [laugh].

R: I couldn't imagine that anybody would like that.

I: Okay, Mhh. But, so you think for certain people, like, so if you would consult, for example, a diagnostic support AI or whatever it's called, you think for some patients they would also not lose trust, but like it would raise questions for some patients regarding your own competency. So this thing you mentioned with: ‘Oh, why does he have to consult this?’ Or do you think they would be like: ‘Okay, this is unnecessary?’

R: I think, it's only some speculations for now because I wasn't in that situation before, but I could imagine that it could happen somewhere, especially like when you have like, older patients, you have known since 30 years. And I think it also depends on the explanation you give when you say: ‘Okay, I'm not sure about maybe some new symptoms you got. That might have several reasons that might be complicated because you're already taking like a lot of different medication for different diseases. Things, like I didn't like you say that like when you have like more than five different substances, it might be difficult to, yeah, to [unclear] with the… the side effects. So, the more medication a person gets, the more difficult it gets to predict what that does with their body. And I think that's something that maybe AI could be useful for.

I: Okay.

R: And maybe if you explain it, like what you use it for, how you use it, what it does. Then, I think, you could like, yeah […] then people would also understand why you're using it and maybe like it, but I think it depends on the person and it doesn't replace you.

I: Okay, Mhh, Okay. So, you would say as long as it's, so to say, well explained and as long as it's very sort of in a very isolated, for a very isolated purpose. So, without the cameras and explain everything, but it's like: ‘Okay, this is why I use it for this specific task’. You think it would be…

R: I think it could be accepted. Yeah. Yeah.

I: Okay. Okay.

R: But of course, when you can't add all the data you would maybe need to see if anything changes.

I: Okay. Okay. And earlier, you said that you see a potential challenge or risk in this in terms of people maybe like patients relying too much on it or like putting in their symptoms and being like: ’Hey, this is it. I rather consult this than going to my GP’, because it might be more accessible or something like that. Are there any other sort of challenges you see or concerns you have?

R: I think that's all about the speculation that I mentioned, but I was also once […] I was thinking about like what happens when this technology gets really good at diagnosing diseases, etc. And then, the people who have… insurances or other institutions that have to pay for it say: ‘Okay, yeah, that's a lot cheaper than to have like doctors or other healthcare practitioners working’. So, yeah.

I: Okay.

R: Just let's do AI do that that. I'm afraid of this. I think you can lose people there because they're saying: ‘Okay, I don't trust it. I'm not using it.’

I: Okay.

R: Or on the other hand, maybe they wouldn't tell like all of their, what their problems like if they have like also like problems with their… in their social environment, etc. If you would like tell that to an AI and like replace this when you're replacing this relation between a patient and healthcare provider, which is with a computer. I think that that would be like a really, really bad thing. But honestly, I don't think that that would happen.

I: Okay. Okay. Okay. But also, so also in a way related to this point of: ‘Okay, there are certain components of the human like doctor patient relationship, which are probably like impossible to replicate, and one shouldn’t try to replicate them because like the health insurances or like, yeah, you would see risks in the attempt to replicate it.

R: I would say so. I would say like other specialties, I think when too many people decide to become radiologists now, they might have a hard time in the next 20 years.

I: Okay.

R: With their jobs, but yeah.

I: Okay.

R: I think what you just summarized, I think that affects like most specialties, most settings.

I: Okay. Okay. So especially those specialties, which have this large like human interaction component.

R: Absolutely. Yeah.

I: But because you mentioned that, for example, radiologists, you could imagine that maybe not as many radiologists will be needed in future than are needed now because certain aspects could be automated. Is that also the case in your eyes, for example, for something like surgery, where, yeah, which also at least in my lay perspective lacks the direct human relationship?

R: […] That's a really good question and I'm not an expert for this. I think that there are some, also there's some human perspective because usually you're not only seeing that person when you're performing the surgery, but also like in the ward, checking up before and after surgery. So, I think there’s also a human component on this. But yeah, but […] I don’t know like the technical part, I think that technology could also, yeah, support that. Maybe not replace, but I don't know so much about this, but yeah, I think there's also like already like robot assisted surgery that is used in more and more hospitals. So, I don't know why that shouldn't work automatically in some years, especially when also those machines get great at interpreting imaging.

I: Mhh… Okay.

R: But maybe it also gives more time to doctors or other medical practitioners because everybody is quite busy with doing lots of paperwork, documentation. Yeah. Also, like imaging takes time. So, maybe it gives, also in the end, gives more time for those talks that people need. And I think most patients need more than they get at the moment.

I: Mhh… Okay. Som in a way that AI assisted work could free up time of the medical professionals for their interactions with patients.

R: Yeah, exactly.

I: This human component, which couldn't be replaced probably.

R: That's what I wanted to say.

I: Okay. Okay. No, that's a really interesting point. Okay. So that certain non-human components could be automated, so to say.

R: Yeah.

I: Okay. You mentioned earlier with regards to your own experience at your office that you really like find very useful the new, like the digitalized systems of record keeping and things like that, but you also have the feeling there are certain risks of relying on it. Is that also something you would sort of be (eeh) concerned about in the context of AI methods?

R: I think there should always be a backup option somehow. If, I don't know, some weird phishing emails is opened at the hospital and suddenly they couldn't do surgery anymore because maybe in 50 years doctors don't, or like surgeons (laughs) don't want to do surgery anymore because it's the robot who does it. What would you do then? So, I think there should like always be some kind of a backup you could work with if there's something is hacked or if there's electricity or something like that. It happens rarely, but it happens. But I think at the moment it's also difficult if there would be like, okay, hospitals have emergency energy supply. But yeah, if somehow something happens that this technology is interrupted, you should be able to continue working.

I: (ehem) Okay… (pause) Yeah, no, it sounds really, yeah, it's definitely an important component or I can imagine it being a key component.

R: And like, people, oh yeah, I was just thinking about that point (crosstalk)

I: Ya ya please

R: People like who are asking for you to use that, if they have maybe some wearables, some smartwatch or something like that, and if those technology have had to diagnose maybe some disease that we wouldn't have found in another way. And like maybe in the end, how patients save lives. I think that's great. I think people are more and more integrating this into their lives. It's always a question of data safety if you want technicals, it's not the medical system that gets these data, but big technology companies. (ehem) So I think that's a large question, but I think it has also its useful sides. I mean, I don't own something like that (laughs) because I'm a little assistant. I'm giving my data about my body to, I don't know, Google.

I: Yeah.

R: But yeah.

I: (ehem)

R: Yeah. It's always two sides with those things.

I: Yeah. Okay. Okay. So also, this data protection aspects in the context of AI.

R: Yeah, absolutely. This data shouldn't be used for something else. I would say so. I don't know how this is possible, how far this is possible, but I think it's a quite relevant thing.

I: (ehem)

R: Yeah

I: And there you would, like, whose responsibility would you say this is? More towards like a policymaking or towards the user or towards the companies? Or like, where do you see the actionable consequences there?

R: I would say it's with policy makers. Because as a user, you can't be aware of all those conditions you're using a product with. And I think that should be made, that should also be made clear by the companies. I wouldn't trust companies (laughs) to regulate it in a way that, in a way that empowers the patient, let's say. (hmm)) But I think there are many that would use it in the way that they can, like, make as much as possible.

I: Yeah

R: So, I definitely would see that with policy.

I: Okay.

R: And I think that also makes it complicated (laughs) It's necessary.

I: And one more question regarding your interactions with colleagues. Like, have you, is this a topic of conversation? Like, is AI a topic of conversation with your peers or with other medical professionals?

I: Not regularly. It's something I'm still a little more than, oh, I heard of that. I heard of this, that we're talking about. And we had some conversations that basically mirrored what we now discussed so far.

R: Okay.

I: There are some that are more like (…) they say, oh, yeah, that's great. I want to use that. I want to try it. There are also some colleagues that say, oh, no, I would never use it. Like, I'm working together. I'm doing this together with the patient. And it's not like, yeah.

R: Okay.

I: Yeah. So.

R: Okay.

I: ehem

R: But it hasn't been like long conversations and discussions. Have you heard of that? Oh, yeah. Okay. Like five minutes (laughs)

I: Yeah. Okay. Okay. And would you say there's any sort of pattern you observe with regards to who is more open towards it or who is more critical about it or sceptical?

R: I think there are like (..) I think there are especially like some younger, like technology liking people that are really part of that. I think there are like many positions somewhere in the middle ground. It's not that. And there are also like some. I think it's especially those who have been like working for decades and say, okay, yeah, that would like change how I'm working. And I like I've got patients I know for so much time. They're quite happy with me. And I'm not sure if that would have like we would have that connection to each other. (ehem) We'd have used if I had to have used AI. So, yeah, I think that's basically both sides.

I: Okay.

R: And the middle ground. But there are also like some different opinions on ultrasound already. So... (laughs)

I: Yeah. (laughs)

R: Not in general, like nobody's saying, oh, I'm not using that. But like different opinions on special check-ups that could be like screening or not. So, for example.

I: Okay. Yeah, of course.

R: But of course, AI is something completely different.

I: Yeah.

R: And to this. Relationship. Like (...)

I: Maybe it's one just final question (ehm) So you described this general. Okay. Yeah. There might be a way of integrating it also for you in terms of like in a certain like limited diagnostic context (ehm). How... how would you learn about this? Like where would you imagine like learning how to use it? Is it something you teach yourself or is something that you would want to see like a broader capacity building programs? (ehm)

R: Like when I would start to use that, I would like to know that it was tested a lot (laughs) and that it really works. And I think that every technology is only as good as the user in front of it. So it's like an extensive training. Somehow, I wouldn't just teach that myself because like when you just put stuff into it, that technology doesn't work with maybe like it. And that might have a negative impact on diagnosis and in the end of how the patient is treated.

I: Ehem

R: So, I think that there needs to be a lot of training on that and that it has to be like tested before and also that you really know about the limitations.

I: Okay

R: And that's something super important that there should be information on that for medical practitioners, but also for patients that they know about these and limitations of technology.

I: Okay. So, for you to use something like this, the requirement would be like very comprehensive testing beforehand and very comprehensive information. What can it do? What can it not do? Things like this. And also, some form of certified training program for you to feel comfortable using it.

R: Definitely. And also, maybe some like some certified program where you are either tested or where somebody maybe supervises you when start working in your environment.

I: Yeah. Okay. Okay. What type of body, like a training body, could you imagine? Like what would the organizations be that could offer such a training?

R: Like usually trainings are organized by the like the state medical boards. I think that would be something for those who are together with, yeah, of course, those who implement those technologies. I think there should be like, it shouldn't just be done by companies, but by like some certification, some public bodies.

I: Okay. Okay. Thank you. (…) That's it with regards to questions from my side. Is there anything that we haven't discussed so far?

R: I think I always edit something when it came into my mind. So, I'm sorry if it's like...

I: No, this is perfect.

R: This is like a conversation. Yeah.

I: No, this is great. Thank you so much for your, (laughs) for your comprehensive, like for sharing your perspective. (ehm) Are there otherwise any open questions that you have for me?

R: (hmmm) Not yet, honestly. Are you working together with some artificial intelligence company or something like that?

I: No, not right now. Actually, we are more trying to evaluate the general perspective of medical practitioners and where they see the potential and where they also see challenges or have concerns, sort of given evidence-based, (eee), yeah, recommendation or insights into this, whilst like being completely independent from larger companies (ehm) that are developing something like this.

R: That sounds like super interesting work.

I: (laughs)

R: Looking forward to read about it.

I: Thank you. Yeah. And always feel free to reach out if something comes to your mind. Yeah. We are very, very happy to hear from you.

R: Okay. Yeah. Thanks a lot.

I: Thank you so much. (laughs). Bye.

R: Bye.

Original interview guide

[Welcoming the participant, thanking them for their time, introducing the study and purpose]

1. To start off, could you please tell me about your day to day work?

2. What do you think about digital technologies in clinical practice?

3. To move even further, what is your impression of Artificial Intelligence? What have you heard about it?

4. Do you already use AI?
   a. In your work, in private life?
   b. What does this look like?
   c. How does it currently influence your career?
   d. Interactions with colleagues, interactions with patients, your own work, how you see yourself etc.?

5. Have you heard about AI being used by your clinical colleagues? Others in the medical field?

6. Where do you see future opportunities related to AI in your day to day work life? Where do you see challenges?

7. How do you in future see it influence your career? In what ways/domains?

8. Within which clinical domain do you see tremendous opportunity related to AI?
   a. What might be challenges to unlock the full potential for AI in these domains?

9. Where (within which clinical domain) do you see AI serving less of a purpose or having less relevance?
   a. What would have to change/what would be required so that AI could serve a purpose there?

10. Have you noticed any distinctions in terms of who within your clinical environment seems to be more or less open to the AI? What do you think underpins this perspective?

11. You mentioned earlier doing XXX/imagining doing YYY. Where have you learned about this?
    a. How could training for this look like?
    b. What infrastructure would be required?

12. I have reached the end of my questions; is there anything you would like to add?
    a. Do you have any questions for me?

Upload prompts based on *ChatGPT Splitter*

Prompt 1

Act like a document/text loader until you load and remember content of the next text/s or document/s. There might be multiple files, each file is marked by name in the format ### DOCUMENT NAME. I will send you them by chunks. Each chunk start will be noted as [START CHUNK x/TOTAL], and end of this chunk will be noted as [END CHUNK x/TOTAL], where x is number of current chunk and TOTAL is number of all chunks I will send you. I will send you multiple messages with chunks, for each message just reply OK: [CHUNK x/TOTAL], don't reply anything else, don't explain the text! Let's begin: [START CHUNK 1/6] ### file.txt ### I: Hi, ahem, yeah thank you again for participating in today's interview. Ahem, maybe to start off, could you please just tell me a little bit about your, your own day-to-day clinical work? R: Ok, Yeah, ahem, thanks a lot for having me. Ahem … Usually, ahem our, or my doctor's office is opened from Monday to Friday and it's almost every day, ahem, the same. Usually, in the mornings, ahem we have a patient consultation, ahem, some, ahem have chronic diseases, some have something … more urgent. Ahem, and, yea different sorts of patients, ahem, different backgrounds, younger people, older people, ahem … yes from the area here. So, also many patients that, ahem, I know for a longer time now, … like for … a few years after I started. Ahem, yes, in the afternoon, on some days, we have it as well. We also do some, ahem, visits at patients' homes … and, ahem, we also do some examinations, as for example, ultrasound … examinations that are planned in the afternoon with patients that, for example, come for regular checks, check-ups. I: Okay, okay, thank you so much. Just … just for me to understand, ahem, is it like in, in your practice, is it just you or are you several doctors sharing one office, so to say? R: Yea, we are several doctors, ahem. I'm still, ahem doing my specialization, so I also have a supervisor with whom I'm meeting on a regular basis, but who I can also consult if I'm not sure what's the case. Ahem, but yeah, we are like five … five medical doctors working together in the practice. I: Okay, and all of you have a, ahem … the specialization in, like, just as a GP? Or do you have diverse backgrounds? R: Yeah, ahem, most, ahem, one has also a specialization in internal medicine, but it's like working more or less as a general practitioner … and, ahem, some have also some, ahem, additional specializations as … emergency medicine, ahem … or something. I: Okay, okay. So… R: Generally, we are all working as general, … or all in this office are working as general practitioners. I: Okay, okay, thank you. And, ahem, that means, so if I understood correctly, like in … in general, you have this, like, mixture of patients throughout the week, sometimes in the afternoons you have more … specialized, ahem, like diagnostic procedures planned, ahem, things like that. And then, … ahem, but do you also perform surgeries and these kinds of more complicated procedures or is that usually something where you refer your patients to other? R: It's something we refer … refer patients for, ahem, to … to other clinics or offices, yes. I: Okay, okay. R: When … Something that needs the expertise of another specialty. I: Okay, okay, thank you. Great. Ahem, and your, your patient base, so you said you have basically the full spectrum from rather young patients to rather old patients and from chronic complicated cases and also more day to day, like, smaller emergencies, so to say. R: Yeah, exactly. There are some patients that have been coming to this office for … 10 or 20 years now. Of course, I'm not there for this long time, but … all the colleagues know … know them quite well and I also had a chance to, to get to know … several of them during the last … two, three years. Ahem, but yeah, for the day to day work, people having a cold or something similar ... Yeah. I: Ah that's really nice to have this time to really form a relationship with the patients, like basically over, yes, over many years. Ahem, that sounds really nice. R: [laughs] I: [laughs]. Thank you. Ahem … So maybe to, to … ahem, move on to the next questions. Ahem, in general, what is your perspective or what do you think about digital technologies in clinical work or in clinical practice? R: I think it depends. I think there are many new tools that … ahem, can be used in … ahem, or that could work, make easier or more efficient. Ahem, for example, … ahem, digital,

ahem, like we have like this … system in our, ahem office where we can … where we have all the patient's data, all ahem … information we put into it, all examinations that were done. We could check that. I think there's also like, ahem … there are more things that could be done, but I'm not yet, especially Germany, ahem, where it always takes a long time until technology is adopted [laughs]. … Ahem, so yeah, I think there are things that can … make work easier, but you also depend on it. So if one day this … system we use here in the office is not working, ahem, that makes things difficult [laughs] because everything, ahem, is based on that. And … ahem … but yeah, I think there are chances, but there are also, like … challenges, ahem, in clinical practice. I: Okay, yes. So, if I understood it correctly, there, like, the way it's currently implemented at your own practice, ahem, you mainly use it for, like, record keeping and for, ahem, basically, yea, structuring your records. Ahem, and there are certain things if I understood it correctly, where you would say, … well, you would like to do, or you would be open to implement it more strongly, but there are certain ways in which maybe regulations are like on a larger, … like you said on a national level, certain things might not yet be possible? R: Yeah, especially connecting … like, for example, ahem, office with hospitals, that we can send information to each other more easily and … yeah. Stuff like that. I: Ah Okay. Okay. … Okay. R: And another way I have been in contact with that, it's like also some patients having those watches, those smart watches. I: Hmm, okay. R: Some also like … ahem, checking for example your, your … ahem, yea some basic information on … like your heartbeat or something like that. Ahem … I don't know how that exactly works, honestly. I should, but [laughs] … maybe I should teach myself more about that. But yeah, I know that some people are tracking … are tracking that, ahem, using this. And I've also read about cases … cases, ahem, where … where those watches, ahem … Warn patients about like, say, ahem rhythm anomalies, but yeah. … But I never had that case, ahem … seen. Like, I've never seen that myself so far. I: Okay. Okay. But, ahem, were there instances where … you already have used data from, from smart watches in your own interactions with patients? R: No, no, I've never used that so far. I: Okay. R: Like I have some patients who talked … about that, bit for them them, they were healthy. So, ahem … in my view. So, there was nothing, ahem, yeah … like no abnormal, ahem, like, yeah. I: Okay. Okay. R: Nothing bad [laughs]. I: Okay. So that data, in a way they have it available through their … watches or whatever, ahem, but it doesn't yet play any role in your … in your day to day work, so to say? They mentioned about it, but it's not integrated? R: Not yet. Not yet [laughs]. I: Okay. Okay. R: It's interesting. I think it's super, it's interesting, but yeah. … I haven't had the chance to … to learn how to make use of it, … but I'm interested in to it. So … I: Yeah. Okay. And also, your other colleagues, like … especially the colleagues within your office … like they are also not yet working with things like that? R: Hmm … not, not people I have direct contact to, like we had some discussions about it. I also heard about some applications in … in clinical work, but not, ahem, in my, in my close environment. I: Okay. Okay. Thank you. … Maybe moving on to this broader topic of, ahem, artificial intelligence. R: Hmmm: I: Ahem, what is your impression of that and … what have you heard about, yeah, this buzzword of artificial intelligence, so to say? R: In general, or, ahem, only like in the field of medicine? I: Ahem, I would say now to start off in general, and then we can move on to the clinical context. R: Okay. … In general, it feels like, ahem … everybody's talking about ChatGPT [laughs]. I: Hmm R: But … yeah, I … I have to say, ahem, I'm not sure about my feelings on i [END CHUNK 1/6] Reply with OK: [CHUNK x/TOTAL], don't reply anything else, don't explain the text!

Prompt 2

[START CHUNK 2/6] t. I think it can help a lot, but … I think, like, when it gets possible to … to, ahem, to fake interviews, things that people have said … fake videos, maybe also fake scientific data, … it makes … Yea, I'm a little afraid that this might make things complicated or … even, like … could be used by … I don't know … political actors or some, something like that to, for … many, many things that might not be … might have, ahem very negative impact. So yeah, that's something I don't know so much about, but … I'm a little afraid of that those … those …. technical tools one can use them in a quite bad way [laughs]. I: Yeah. Okay. Okay. R: But yeah, generally I think they're also, like, positive perspectives. I: Okay. Okay. Okay. And …. So, this, these concerns that you described, they … basically apply to the general, … like, to everyday life, so to say, or all aspects of life? Or are there specific aspects that you would apply them to? R: I would say, ahem, to all aspects of life. Like, especially I, I have been thinking about it like in this … about this, in this political field, but … I think, yeah, they're also like, … I think we will have, at some point, it will be possible to have artificial intelligence … in so many different … ahem, yea, facets of your life. So … yeah. I: Okay. Okay. R: Also, medicine. Yeah [laughs]. I: Yeah. Ahem, yes, that would have been [laughs] that would have been my next question. So, the … is there already, like, have you already heard about it in the context of clinical work or of medicine? R: There are like two things I've heard of. One … are, ahem, softwares helping, for example, analyzing … ahem, basically data from examinations, like for example, ECG or, ahem … ahem, or, yeah, other, like similar, similar things, analyzing x-rays or … ahem … yeah, other, ahem ... Other, other imaging techniques. Ahem, …. yeah. That's like the two things I've basically heard of … that there are studies are, or, also already in institutions using those technologies who are getting, … who were in some cases better than human, ahem, human, ahem, doctors in analyzing. Yeah. I: Okay. Wow. Okay. R: For Imaging processes. I: And then analyzing, so to say, to reach to … to a more specific diagnosis or, or more specific recommendations than a human would in this context? R: I'm honestly not sure. It's quite some time ago, but I think it's, it's less recommendations, but more, like, diagnosis. Ahem, or for example, locating something, or like … seeing small ECG changes that … maybe can be overlooked by, by … by other, like, people, ahem [inaudible]. I: Okay. Okay. Yeah. It's really interesting. Yeah. R: But I'm no, I'm not an expert on that. So … [laughs] I: Yes. So, you said, sorry, if I … I didn't catch it, you said there are two, two sort of fields that you already, ahem, heard about it. So, one being this, ahem … broader, like, analysis of examination data? And what was the second one? Sorry. R: Yeah. That were decision support system, I think it's called decision support system. I: Ok. R: I'm not 100% sure, but yeah, ahem, it's like, ahem, it's also used for diagnosis. And … like, I think there are also already, like, apps on that, that are working with artificial intelligence, ahem, for example. And it's also tested in the clinical field where you put, like, the symptoms. I: Ah. R: And, and I think also, like, data from examinations as, ahem, yeah … I: Mhm. R: As, for example, yeah. Images… or… blood values into… I: Okay. R: That might form like… chances of different diagnosis, maybe finding something that, aehm, we wouldn't have thought about. I: Mhm. R: Yeah, and I think that exists, so far exists, in, like, this field where it's tried out and also already like… apps for…for smartphones that everybody can use. Where you can, like, check your symptoms. I: Okay. R: Yeah. Not sure how that exactly works. And… like… I: [laughs] Okay. Okay. Yeah, and have you, in any way, already used any of these… like, or artificial intelligence in any form in your work? R: Ehm, not in my work. I tried out one of these apps [laughs] once, aehm, but that's quite some time ago. I: Okay. R: Yeah. I: The diagnostics, like where you put symptoms and things like that… R: Ehm, and quite some time ago, I don't remember…remember too much. I thought, oh yeah, that's interesting. But then I didn't follow up. I: Okay. And what was your experience with it or what did it say so to say? [laughs] R: As mentioned it was already some… some time ago. Ehm, I'm not sure, like… like it showed like five diagnosis rates that, okay, yeah, that might be possible… [both laugh] Okay. I just put that. Ehm, yeah and compared to when you Google your symptoms, it doesn't say like, oh yeah, you might have this very bad disease and this and this, but it's like also working a little more. I: Aah. R: I think that's really real probabilities and nothing where a tumour that you might have… just when you only have headache or something. I: Okay. R: So, ehm…

I: So, the results are less drastic or like unlikely drastic. R: Yeah. Yeah. But I think, I…, it also showed them. So that's possible… You should maybe go and check, but ehm yeah, don't like… it didn't make you as much… or like, I felt that when I would read it both, it wouldn't make me like panic as I would just Google it and be like, oh my God. I: Okay. Okay. Is that something?... [both talk at the same time] Sorry… R: It's quite some, it was quite some time ago and I was still studying and yeah. Ehm. And so, I don't remember too much, but that's what I, but I think that I remember quite some time ago. I: Okay. Is that something that already comes up in your interactions with your patients? Like, do people come to you and talk about, aehm, having used AI in any form or expecting you to use it in any way? R: Ehm… No, not yet. People who Google, this patients who Google their symptoms or use Google or some other search engine, ehm, for looking up their symptoms, yes, that happens, but, aehm, I… don't remember that anybody that would… using AI or some, at least they didn't talk some… or… and nobody expected to use it. I: Okay. Okay. R: I once had a discussion with a patient who asked if… if we know about… like who just read some newspaper article about that. Aehm. That was that. I: Okay. And, but that patient also was more just interested in hearing your opinion about it? R: Yeah. Yeah. Yeah. So, no… no… no expectations yet. I think most of it is still on file. I: Okay. Okay. Okay. But based on your experience, aehm, the, like it might actually be less concerning if people would put their symptoms into… these kinds of AI systems, then just Googling it and, and come to you being concerned of having a very severe disease? R: I think [laughs] it's… I think if you have symptoms and go for a check-up, it's always good… [both laugh] Yeah. It might be good that they're not that concerned, but it would be good if like, aehm, anyways. Aeeehm… But I… I'm not sure about this. Like… like this is one… one like point or one layer, but I also… think that I shouldn't make people think or that the fact that they, people, should not think… the fact that they have checked their like symptoms or something else or feeling with an AI that, that yeah… that they don't have to go to like some medical practitioner to check it. Aehm, so… aehm, that's it. Oh yeah. Aehm, I… I only use that app now. It replaces all my apps. I: Ah okay… R: Because I think that medical practice are like… are like, yeah, our job is more than just like have a list of symptoms and there's also like a person behind it. And you see that person, aehm, how the person presents themself, aehm, how he or she behaves, how… yeah, you might also think it's just like, how do you feel? And that's… that's stuff that I think that's difficult to be replaced by an AI. Aehm… So… I think that like… aehm, only talking [END CHUNK 2/6] Reply with OK: [CHUNK x/TOTAL], don't reply anything else, don't explain the text!

<u>Prompt 3</u>

[START CHUNK 3/6] about diagnosis now, not about… [laughs] about recommendations or treatment. I think that's something completely different, where it's, aehm, like, where I'm not sure how we could use AI there or what could be there because they, I think they really need that, aehm… like a relationship somehow, especially with chronically ill patients. Aehm… They need something, somebody to trust. And like, it's trust always goes first. So yeah. Aehm… So, I'm not sure how it would work with that. But I think, aeehm, we were still at diagnosis, so... And also, like psychological, psycho… aehm, is psychosomatic, also the word in English? I: Mhm. R: Aehm… Yeah. Symptoms of… and their connections with aehm, with the body that I think, I'm not sure how this can be caught by an AI. Maybe it can. I… Aehm… And also, patients like describe or go share, aehm, symptoms maybe differently. They might have the same disease, but for one person… okay. People have different symptoms, but people also like say, describe… aehm… for example, a pain differently. I think therefore it's, I'm not sure if you can just put it on a scale on AI and say, okay, yeah, that's, that works perfectly. You don't need a doctor here anymore or somebody else… I: Okay. Okay. Okay. So, in that sense, would you say that… you see… like you will have experienced or you have heard about it and you could also imagine using it in the context of like as diagnostic support, so to say? R: Yeah, I think like that's the point. Like I wouldn't say that it kept like replace like… aehm… diagnosis… and like, aehm, you're really talking to people, but it could support it. Maybe when you can enter symptoms there and maybe it says, oh yeah. When you don't know what that could be. And maybe I heard of it, like I heard that those systems are simply used at aehm… ambulan… aehm… clinics for rare diseases. I: Mhm. R: So, maybe it finds something that you haven't been considering because maybe you just didn't know… because there's like so many rare diseases. I'm not sure if anybody could know all of them… with… aehm… symptoms and recommendations. Aehm, so, I think therefore it could really be helpful as well, but… I think it couldn't replace. [laughs] I: Yeah. Yeah. Okay. R: …consultation, but it could support. And I think that's really… therefore… I found that super interesting. I: Mhm. Mhm. Yeah, no, definitely. R: Or maybe saying, oh, that doesn't fit here. Maybe it's like… aehm… when you're saying you, so you can have two diseases at the same time…. Aehm… Oh yeah. But I'm not worrying about both… I: Okay. So, you described that like one of the… the key opportunities related to AI now in your work, you would see in this like diagnostic support or like… maybe identifying certain patterns that might be… that otherwise might be overlooked. If I understood correctly…? R: I would… Yeah. Yeah. That's what I would say. I: Okay. R: For the diagnostics. [laughs] I: Mhm. Are there any other like opportunities, aehm, you would see? Like now or in future also looking at how the… like potential developments in the field? R: Like… for treatment… I'm not so sure on that. I think maybe it can look for studies, recommendations... Aehm… There are the official recommendations for several diseases. Maybe it could help, especially in the case of rare diseases at some point. But also in this case, I think it doesn't… repl… it's not really possible to replace a physician there or other medical practitioners. Like… That's also the case. As I mentioned before, you need connection to the person. Or at least I would say that you need that. Aehm… A person who like doesn't see you like as members, but as the person you are, knows your social environment… And that's also something super important and… I'm not sure how like… the intelligence should deal with that… I: yeah... R: ehm… Also, we are living in an age of shared decision making. I'm not sure how… I could try to make decisions with a patient together and support the patient in making decisions. How that would work out without that personal connection. And also… like after making a decision, you're doing checks on your patient. How… aehm… maybe also how this person is like maybe changing in her feelings or like in her presentation or behavior. I'm not sure if a… AI could grasp that when you have been seeing a patient for… over years. And see how they are behaving, how they are talking, how they are maybe feeling… I: Mhm. R: I think it wouldn't be possible to replace that… Aehm, and also like you can… maybe that would be something like interaction of medication or something like that. Something I could see that, but also like isn't just describing the medication. It's also communicating that it's… how it's important to take it in a certain way. Because I think there are studies on it that a lot of patients, almost half, doesn't take their, aehm, medication as prescribed. Aehm… As I would

myself. Yeah. [Both laugh] But also if I'm sick, I'm also not taking everything as I should. I: Mhmmmm. R: So, I'm also lying to this 50% that doesn’t take it. [both laugh] I think it's important to communicate why and how and where you could maybe leave out once. Aehm, also like forwarding the advantages and disadvantages of medication. Aehm… or like of a combination of medications. Maybe that's the part where I could support too… But… aehm… coming to side effects and how a patient… aehm… experiences side effects of medication? I'm not sure if things like that are possible to grasp by an AI,, but… but, yeah. Maybe it could like help on this. Okay, yeah… this medication might have these side effects. And then also then we're coming back to diagnostics. I: Mhm. [laughs] R: It’s connected. [laughs] I: Okay, okay. So, you mentioned that you as a practitioner who potentially has interacted with the same patient over years and of course has collected all this information and built this relationship over time. Do you think there that potentially an AI if it would also have sort of the same sort of basis of information? Like to imagine that the AI has exactly the same number of interactions or the number of varying descriptions of their symptoms and things like that. So, do you think with having a bigger database for an AI, this could also make it more competent in interacting with the patient? Or would you say, there's just always something that this kind of algorithm or AI could not grasp, no matter how much data it has? R: A really good question indeed. I'm not sure what AI can do or what it will be able to do in 10, 20 years. So maybe, it's possible if you have cameras everywhere that the AI can analyze a patient's behavior, their face, how they're talking. I'm sure that that will be possible at some point. Another question is if people want that. I: Okay. R: So, I think a trustful relationship between patient and health practitioner is important for both. As a patient, I'm also happy when I can go to another - when I have something, and I can go to a doctor with a specialty maybe. Or, also not just be treating myself, but when I can go there and have a trustful conversation. And I know that that's only that person listening to me and no computer that analyzes everything. And that person that maybe tries to understand me and see me and not only to analyze. So, I think, I wouldn't want that as a patient. I: Mhhh R: I wouldn't want that as a practitioner, but... yeah… I: Yeah, this trusted relationship, it's a really relevant point, of course. Could you imagine cases where you yourself would have maybe more trust into an AI in a clinical setting than in a human doctor? R: I think it always depends on the human doctor [laughs]. I could imagine that in some cases where I'm not really sure about symptoms and explanations I have, how that fits together and there are some things that are a little[...] Maybe they're strange. […] I would say, okay, maybe I’ll see AI. Maybe it's [END CHUNK 3/6] Reply with OK: [CHUNK x/TOTAL], don't reply anything else, don't explain the text!

Prompt 4

[START CHUNK 4/6] something that doesn't appear so often. Maybe some rare side effects – it doesn't have to be a rare disease, but some rare side effects of a standard medication. That happens. I think in those cases, yeah, I think there are situations where I would say: ‘Okay, maybe let's check’. Maybe not replacing the doctor, but like, working together. I: Okay, okay: And how do you think your patients would react if you would integrate, for example, for the diagnostic component, integrate AI into your interactions with them or into your consultations? R: Ehm, I think [laughs] there would be mixed reactions. I: Okay. R: I think there would be some patients that would say: ‘Oh, cool, he's checking up with that AI. Maybe also to not overlook something.’ Maybe there are some other patients who would say: ‘Okay, what are you doing? Do you have to google and look up everything?’ in the interaction. I: Okay. R: So, I think there would be mixed reactions. I think that also not at this point, not everybody knows about it. There's not so much knowledge. There's not so much known about how these technologies work. Ehm, so, yeah, I'm not sure about, I think there would be mixed reactions. I: Okay. R: But I think when I would like install cameras everywhere and say: ‘Oh yeah, that's just eye tracking, you have to talk with it. I'm just sitting here`. I&R: [laugh]. R: I couldn't imagine that anybody would like that. I: Okay, Mhh. But, so you think for certain people, like, so if you would consult, for example, a diagnostic support AI or whatever it's called, you think for some patients they would also not lose trust, but like it would raise questions for some patients regarding your own competency. So this thing you mentioned with: ‘Oh, why does he have to consult this?’ Or do you think they would be like: ‘Okay, this is unnecessary?’ R: I think, it's only some speculations for now because I wasn't in that situation before, but I could imagine that it could happen somewhere, especially like when you have like, older patients, you have known since 30 years. And I think it also depends on the explanation you give when you say: ‘Okay, I'm not sure about maybe some new symptoms you got. That might have several reasons that might be complicated because you're already taking like a lot of different medication for different diseases. Things, like I didn't like you say that like when you have like more than five different substances, it might be difficult to, yeah, to [unclear] with the… the side effects. So, the more medication a person gets, the more difficult it gets to predict what that does with their body. And I think that's something that maybe AI could be useful for. I: Okay. R: And maybe if you explain it, like what you use it for, how you use it, what it does. Then, I think, you could like, yeah […] then people would also understand why you're using it and maybe like it, but I think it depends on the person and it doesn't replace you. I: Okay, Mhh, Okay. So, you would say as long as it's, so to say, well explained and as long as it's very sort of in a very isolated, for a very isolated purpose. So, without the cameras and explain everything, but it's like: ‘Okay, this is why I use it for this specific task’. You think it would be… R: I think it could be accepted. Yeah. Yeah. I: Okay. Okay. R: But of course, when you can't add all the data you would maybe need to see if anything changes. I: Okay. Okay. And earlier, you said that you see a potential challenge or risk in this in terms of people maybe like patients relying too much on it or like putting in their symptoms and being like: ’Hey, this is it. I rather consult this than going to my GP’, because it might be more accessible or something like that. Are there any other sort of challenges you see or concerns you have? R: I think that's all about the speculation that I mentioned, but I was also once […] I was thinking about like what happens when this technology gets really good at diagnosing diseases, etc. And then, the people who have… insurances or other institutions that have to pay for it say: ‘Okay, yeah, that's a lot cheaper than to have like doctors or other healthcare practitioners working’. So, yeah. I: Okay. R: Just let's do AI do that that. I'm afraid of this. I think you can lose people there because they're saying: ‘Okay, I don't trust it. I'm not using it.’ I: Okay. R: Or on the other hand, maybe they wouldn't tell like all of their, what their problems like if they have like also like problems with their… in their social environment, etc. If you would like tell that to an AI and like replace this when you're replacing this relation between a patient and healthcare provider, which is with a computer. I think that that would be like a really, really bad thing. But honestly, I don't think that that would happen. I: Okay. Okay. Okay. But also, so also in a way related to this point of: ‘Okay, there are certain components of the human like doctor patient relationship, which are probably like impossible to replicate, and one shouldn’t try

to replicate them because like the health insurances or like, yeah, you would see risks in the attempt to replicate it. R: I would say so. I would say like other specialties, I think when too many people decide to become radiologists now, they might have a hard time in the next 20 years. I: Okay. R: With their jobs, but yeah. I: Okay. R: I think what you just summarized, I think that affects like most specialties, most settings. I: Okay. Okay. So especially those specialties, which have this large like human interaction component. R: Absolutely. Yeah. I: But because you mentioned that, for example, radiologists, you could imagine that maybe not as many radiologists will be needed in future than are needed now because certain aspects could be automated. Is that also the case in your eyes, for example, for something like surgery, where, yeah, which also at least in my lay perspective lacks the direct human relationship? R: […] That's a really good question and I'm not an expert for this. I think that there are some, also there's some human perspective because usually you're not only seeing that person when you're performing the surgery, but also like in the ward, checking up before and after surgery. So, I think there’s also a human component on this. But yeah, but […] I don’t know like the technical part, I think that technology could also, yeah, support that. Maybe not replace, but I don't know so much about this, but yeah, I think there's also like already like robot assisted surgery that is used in more and more hospitals. So, I don't know why that shouldn't work automatically in some years, especially when also those machines get great at interpreting imaging. I: Mhh… Okay. R: But maybe it also gives more time to doctors or other medical practitioners because everybody is quite busy with doing lots of paperwork, documentation. Yeah. Also, like imaging takes time. So, maybe it gives, also in the end, gives more time for those talks that people need. And I think most patients need more than they get at the moment. I: Mhh… Okay. Som in a way that AI assisted work could free up time of the medical professionals for their interactions with patients. R: Yeah, exactly. I: This human component, which couldn't be replaced probably. R: That's what I wanted to say. I: Okay. Okay. No, that's a really interesting point. Okay. So that certain non-human components could be automated, so to say. R: Yeah. I: Okay. You mentioned earlier with regards to your own experience at your office that you really like find very useful the new, like the digitalized systems of record keeping and things like that, but you also have the feeling there are certain risks of relying on it. Is that also something you would sort of be (eeh) concerned about in the context of AI methods? R: I think there should always be a backup option so [END CHUNK 4/6] Reply with OK: [CHUNK x/TOTAL], don't reply anything else, don't explain the text!

Prompt 5

[START CHUNK 5/6] mehow. If, I don't know, some weird phishing emails is opened at the hospital and suddenly they couldn't do surgery anymore because maybe in 50 years doctors don't, or like surgeons (laughs) don't want to do surgery anymore because it's the robot who does it. What would you do then? So, I think there should like always be some kind of a backup you could work with if there's something is hacked or if there's electricity or something like that. It happens rarely, but it happens. But I think at the moment it's also difficult if there would be like, okay, hospitals have emergency energy supply. But yeah, if somehow something happens that this technology is interrupted, you should be able to continue working. I: (ehem) Okay… (pause) Yeah, no, it sounds really, yeah, it's definitely an important component or I can imagine it being a key component. R: And like, people, oh yeah, I was just thinking about that point (crosstalk) I: Ya ya please R: People like who are asking for you to use that, if they have maybe some wearables, some smartwatch or something like that, and if those technology have had to diagnose maybe some disease that we wouldn't have found in another way. And like maybe in the end, how patients save lives. I think that's great. I think people are more and more integrating this into their lives. It's always a question of data safety if you want technicals, it's not the medical system that gets these data, but big technology companies. (ehem) So I think that's a large question, but I think it has also its useful sides. I mean, I don't own something like that (laughs) because I'm a little assistant. I'm giving my data about my body to, I don't know, Google. I: Yeah. R: But yeah. I: (ehem) R: Yeah. It's always two sides with those things. I: Yeah. Okay. Okay. So also, this data protection aspects in the context of AI. R: Yeah, absolutely. This data shouldn't be used for something else. I would say so. I don't know how this is possible, how far this is possible, but I think it's a quite relevant thing. I: (ehem) R: Yeah I: And there you would, like, whose responsibility would you say this is? More towards like a policymaking or towards the user or towards the companies? Or like, where do you see the actionable consequences there? R: I would say it's with policy makers. Because as a user, you can't be aware of all those conditions you're using a product with. And I think that should be made, that should also be made clear by the companies. I wouldn't trust companies (laughs) to regulate it in a way that, in a way that empowers the patient, let's say. (hmm)) But I think there are many that would use it in the way that they can, like, make as much as possible. I: Yeah R: So, I definitely would see that with policy. I: Okay. R: And I think that also makes it complicated (laughs) It's necessary. I: And one more question regarding your interactions with colleagues. Like, have you, is this a topic of conversation? Like, is AI a topic of conversation with your peers or with other medical professionals? I: Not regularly. It's something I'm still a little more than, oh, I heard of that. I heard of this, that we're talking about. And we had some conversations that basically mirrored what we now discussed so far. R: Okay. I: There are some that are more like (…) they say, oh, yeah, that's great. I want to use that. I want to try it. There are also some colleagues that say, oh, no, I would never use it. Like, I'm working together. I'm doing this together with the patient. And it's not like, yeah. R: Okay. I: Yeah. So. R: Okay. I: ehem R: But it hasn't been like long conversations and discussions. Have you heard of that? Oh, yeah. Okay. Like five minutes (laughs) I: Yeah. Okay. Okay. And would you say there's any sort of pattern you observe with regards to who is more open towards it or who is more critical about it or sceptical? R: I think there are like (..) I think there are especially like some younger, like technology liking people that are really part of that. I think there are like many positions somewhere in the middle ground. It's not that. And there are also like some. I think it's especially those who have been like working for decades and say, okay, yeah, that would like change how I'm working. And I like I've got patients I know for so much time. They're quite happy with me. And I'm not sure if that would have like we would have that connection to each other. (ehem) We'd have used if I had to have used AI. So, yeah, I think that's basically both sides. I: Okay. R: And the middle ground. But there are also like some different opinions on ultrasound already. So... (laughs) I: Yeah. (laughs) R: Not in general, like nobody's saying, oh, I'm not using that. But like different opinions on special check-ups that could be like screening or not. So, for example. I: Okay. Yeah, of course. R: But of course, AI is something completely different. I: Yeah. R: And to this. Relationship. Like (...) I: Maybe it's one just final question (ehm) So you described this general. Okay.

Yeah. There might be a way of integrating it also for you in terms of like in a certain like limited diagnostic context (ehm). How... how would you learn about this? Like where would you imagine like learning how to use it? Is it something you teach yourself or is something that you would want to see like a broader capacity building programs? (ehm) R: Like when I would start to use that, I would like to know that it was tested a lot (laughs) and that it really works. And I think that every technology is only as good as the user in front of it. So it's like an extensive training. Somehow, I wouldn't just teach that myself because like when you just put stuff into it, that technology doesn't work with maybe like it. And that might have a negative impact on diagnosis and in the end of how the patient is treated. I: Ehem R: So, I think that there needs to be a lot of training on that and that it has to be like tested before and also that you really know about the limitations. I: Okay R: And that's something super important that there should be information on that for medical practitioners, but also for patients that they know about these and limitations of technology. I: Okay. So, for you to use something like this, the requirement would be like very comprehensive testing beforehand and very comprehensive information. What can it do? What can it not do? Things like this. And also, some form of certified training program for you to feel comfortable using it. R: Definitely. And also, maybe some like some certified program where you are either tested or where somebody maybe supervises you when start working in your environment. I: Yeah. Okay. Okay. What type of body, like a training body, could you imagine? Like what would the organizations be that could offer such a training? R: Like usually trainings are organized by the like the state medical boards. I think that would be something for those who are together with, yeah, of course, those who implement those technologies. I think there should be like, it shouldn't just be done by companies, but by like some certification, some public bodies. I: Okay. Okay. Thank you. (…) That's it with regards to questions from my side. Is there anything that we haven't discussed so far? R: I think I always edit something when it came into my mind. So, I'm sorry if it's like... I: No, this is perfect. R: This is like a conversation. Yeah. I: No, this is great. Thank you so much for your, (laughs) for your comprehensive, like for sharing your perspective. (ehm) Are there otherwise any open questions that you have for me? R: (hmmm) Not yet, honestly. Are you working together with some artificial intelligence company or something like that? I: No, not right now. Actually, we are more trying to evaluate the general perspective of medical practitioners and where they see [END CHUNK 5/6] Reply with OK: [CHUNK x/TOTAL], don't reply anything else, don't explain the text!

Prompt 6

[START CHUNK 6/6] the potential and where they also see challenges or have concerns, sort of given evidence-based, (eee), yeah, recommendation or insights into this, whilst like being completely independent from larger companies (ehm) that are developing something like this. R: That sounds like super interesting work. I: (laughs) R: Looking forward to read about it. I: Thank you. Yeah. And always feel free to reach out if something comes to your mind. Yeah. We are very, very happy to hear from you. R: Okay. Yeah. Thanks a lot. I: Thank you so much. (laughs). Bye. R: Bye. [END CHUNK 6/6] Reply with OK: [CHUNK x/TOTAL], don't reply anything else, don't explain the text!
