# Supplementary material for: Prompts, Pearls, Imperfections: Comparing ChatGPT and a Human Researcher in Qualitative Data Analysis
Source: Qual Health Res. 2024 May 22;35(9):951–66. doi: 10.1177/10497323241244669 (PMC12202826; doi:10.1177/10497323241244669)
Supplement: Supplemental Material - Prompts, Pearls, Imperfections: Comparing ChatGPT and a Human Researcher in Qualitative Data Analysis [file sj-pdf-3-qhr-10.1177_10497323241244669.pdf]

Prompts, pearls, imperfections; comparing ChatGPT and a human researcher in qualitative data analysis.

Supplemental file 3: Themes and codes identified by the human researcher.

The human analyst followed the phases of Reflexive Thematic Analysis, whereby the transcript was coded as if it was part of a larger dataset and the end goal of analysis would be an academic output. This process included reading and re-reading the transcript, a phase of open coding, and a phase of selective coding which were used to build the initial themes. Themes were then revisited (but were not shared with the entire project team), refined and then shared for comparison with the AI generated output.

Human patient doctor interactions

Unique, patient centred knowledge that can only be gained on face-to-face presentation, and having (in some cases) known the patient for a long time and therefore being able to see when something is wrong (without or with clinical assessment). This is beneficial for both the patient and the doctor. This idea supports or leads into patient doctor relationships that may foster functional and supportive communication environments, again, unique and tailored to that person in that moment.

Codes:

- Symptoms vs patient presentation
- Human understanding of human needs
- Patient doctor relationships
- Illness
- Patient explanations and communication
- Time

Trust

Trust relates to many components of AI and links to knowledge of the patient, and patient interactions. Some patients may welcome the AI because it supports diagnosis, while for others it may reduce or undermine built trust because patients hope doctors have enough professional knowledge without the help of AI. Trust links to fear because if there is limited trust, then there may be a fear of misdiagnosis, misuse of data for purposes unrelated to health, that may also compromise the confidentiality of personal health information.

Codes:

- Trust lost (why using AI)
- Trust gained (because using AI)
- Patient satisfaction (glad they are using AI)
- Information use

- Time
- Data protection
- Companies using data
- In surgery and out of surgery data use
- With patient / without patient data use
- Fear

Diagnostics

This theme relates to positive feelings toward the functional components of AI which may aid doctors in interdepartmental communication, treatment, joint treatment and care decision making

Codes:

- Support
- Working together
- Doctor patient decision making
- Patient observations over time
- Supporting patients with medication
- Recommendations for rare diseases

Knowledge

Knowledge links to trust and human patient doctor interactions because it relates to perceptions of how much people know and therefore, how capable they are of using something like AI. A limited knowledge base and understanding of how AI could be used should be supported by training and skill building.

Codes:

- Colleague perceptions and knowledge level
- Use information
- Limitation information
- Certified information and training

Training

Training was built as a functional code (and was described as an essential action) in response to a knowledge deficit regarding AI that would have to be addressed in order for people to be able to use AI appropriately.

Codes:

- Colleague perceptions and knowledge level
- Use information
- Limitation information
- Certified information and training

AI

This theme links to the general perceptions of AI, AI in medicine and how AI can be used. It links to fear and knowledge because of fake and false information filtering into AI responses.

Codes:

- Fake information
- False information
- Fear
- Examining medical information
- Imaging
- Broad data examinations
- Decision support systems

Digital technology

This theme demonstrates the use, benefits and problems involved with using digital technology in general (and AI specific examples). It shows that there is already a level of engagement with technology that in the future may inform AI, but the engagement is patient specific, useful in some areas and not useful in others. This theme touches on the difference between having capabilities and data, and actually using it to improve hospital efficiency and patient experience and outcomes.

Codes:

- Patient data
- Tech non-adoption
- Makes work easier
- Makes work harder
- Structuring records
- Larger level coordination of data
- Connection of providers
- Personal devices
- How to use data
- Patients have data and use it
- Patients have data and don’t use it
